# Microenvironmental network of clonal CXCL13^+^CD4^+^ T cells and Tregs in pemphigus chronic blisters

**DOI:** 10.1172/JCI166357

**Published:** 2023-12-01

**Authors:** Dawoon Han, A Yeong Lee, Taehee Kim, Ji Young Choi, Mi Yeon Cho, Ahreum Song, Changhyeon Kim, Joon Ho Shim, Hyun Je Kim, Honesty Kim, Hillary Blaize D’Angio, Ryan Preska, Aaron T. Mayer, Miri Kim, Eun-Ji Choi, Tae-Gyun Kim, Eui-Cheol Shin, Kyemyung Park, Do-Young Kim, Soo-Chan Kim, Jong Hoon Kim

**Affiliations:** 1 Department of Dermatology and Cutaneous Biology Research Institute, Gangnam Severance Hospital, Yonsei University College of Medicine, Seoul, South Korea.; 2 Department of Computer Science and Engineering, College of Information and Biotechnology, National Institute of Science and Technology, Ulsan, South Korea.; 3 Department of Dermatology, Samsung Medical Center, Sungkyunkwan University School of Medicine, Seoul, South Korea.; 4 Genome Medicine Institute, Seoul National University College of Medicine, Seoul, South Korea.; 5 Enable Medicine, Menlo Park, California, USA.; 6 Yeouido St. Mary’s Hospital College of Medicine, The Catholic University of Korea, Seoul, South Korea.; 7 Department of Hematology, Asan Medical Center, University of Ulsan College of Medicine, Seoul, South Korea.; 8 Department of Dermatology and Cutaneous Biology Research Institute, Severance Hospital, Yonsei University College of Medicine, Seoul, South Korea.; 9 Laboratory of Immunology and Infectious Diseases, Graduate School of Medical Science and Engineering, Korea Advanced Institute of Science and Technology, Daejeon, South Korea.; 10 Graduate School of Health Science and Technology, College of Information and Biotechnology, Ulsan National Institute of Science and Technology, Ulsan, South Korea.; 11 Department of Dermatology and Cutaneous Biology Research Institute, Yongin Severance Hospital, Yonsei University College of Medicine, Yongin, South Korea.

**Keywords:** Autoimmunity, Dermatology, Autoimmune diseases, Skin, T cells

## Abstract

**BACKGROUND:**

Pemphigus, a rare autoimmune bullous disease mediated by antidesmoglein autoantibodies, can be controlled with systemic medication like rituximab and high-dose systemic corticosteroids combined with immunosuppressants. However, some patients continue to experience chronically recurrent blisters in a specific area and require long-term maintenance systemic therapy.

**METHODS:**

Skin with chronic blisters was obtained from patients with pemphigus. Immunologic properties of the skin were analyzed by immunofluorescence staining, bulk and single-cell RNA and TCR sequencing, and a highly multiplex imaging technique known as CO-Detection by indEXing (CODEX). Functional analyses were performed by flow cytometry and bulk RNA-Seq using peripheral blood from healthy donors. Intralesional corticosteroid was injected into patient skin, and changes in chronically recurrent blisters were observed.

**RESULTS:**

We demonstrated the presence of skin tertiary lymphoid structures (TLSs) with desmoglein-specific B cells in chronic blisters from patients with pemphigus. In the skin TLSs, CD4^+^ T cells predominantly produced CXCL13. These clonally expanded CXCL13^+^CD4^+^ T cells exhibited features of activated Th1-like cells and downregulated genes associated with T cell receptor–mediated signaling. Tregs are in direct contact with CXCL13^+^CD4^+^ memory T cells and increased CXCL13 production of CD4^+^ T cells through IL-2 consumption and TGF-β stimulation. Finally, intralesional corticosteroid injection improved chronic blisters and reduced skin TLSs in patients with pemphigus.

**CONCLUSION:**

Through this study we conclude that skin TLSs are associated with the persistence of chronically recurrent blisters in patients with pemphigus, and the microenvironmental network involving CXCL13^+^CD4^+^ T cells and Tregs within these structures plays an important role in CXCL13 production.

**TRIAL REGISTRATION:**

ClinicalTrials.gov NCT04509570.

**FUNDING:**

This work was supported by National Research Foundation of South Korea (NRF-2021R1C1C1007179) and Korea Drug Development Fund, which is funded by Ministry of Science and ICT; Ministry of Trade, Industry, and Energy; and Ministry of Health and Welfare (grant RS-2022-00165917).

## Graphical abstract

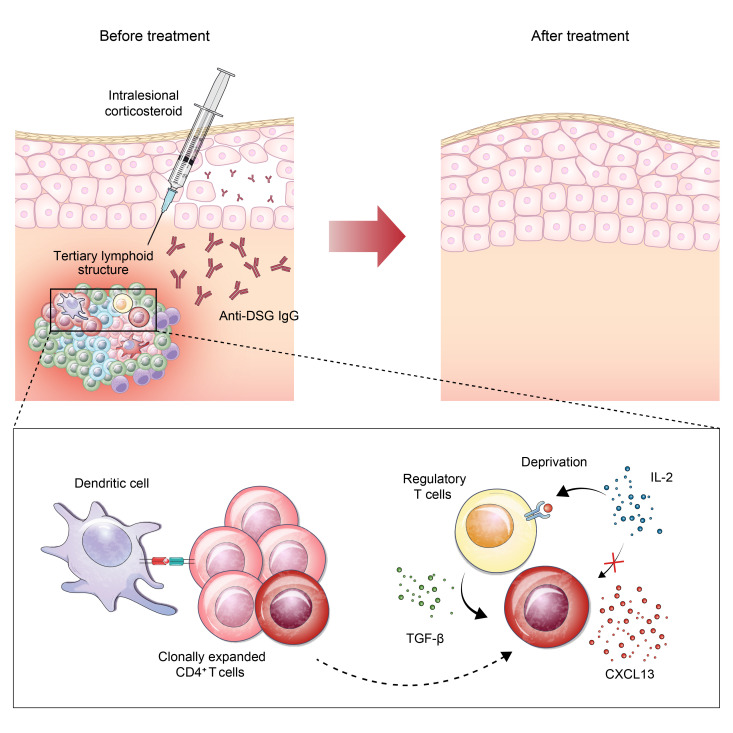

## Introduction

Pemphigus is a rare and life-threatening autoimmune disease that manifests as blisters and erosions on skin and/or mucosa. It is mediated by autoreactive B cells targeting desmoglein 1 (DSG1) and/or DSG3, the transmembrane binding proteins of desmosomes in keratinocytes (1). Anti-DSG autoantibodies cause a loss of cell-cell adhesion in keratinocytes, leading to blistering at mucocutaneous sites (1). Pemphigus is classified as pemphigus vulgaris (PV), pemphigus foliaceus (PF), or paraneoplastic pemphigus (PNP). PV induced by anti-DSG3 autoantibody mainly affects the oral mucosa, whereas PF induced by anti-DSG1 autoantibody presents as superficial blisters on the skin. PNP is known as one of the paraneoplastic autoimmune diseases caused by underlying neoplasms. Following disease control achieved by the use of systemic medications such as rituximab and high-dose systemic corticosteroids with immunosuppressants, treatment can be maintained with low-dose systemic corticosteroids, low-dose immunosuppressants, or no treatment at all (2). However, even with the treatment, some patients may continue to experience chronically recurring blistering lesions on a specific site for several months or more. These lesions, referred to as chronic blisters in this study, rarely disappear despite prolonged use of maintenance systemic treatment. As a result, affected patients may require long-term systemic drug therapy to achieve complete remission.

Tertiary lymphoid structures (TLSs), aggregates of T and B cells, resemble B cell follicles of secondary lymphoid organs (SLOs), such as the spleen and lymph nodes, and have been detected in the peripheral tissues in a variety of inflammatory diseases, including cancer, infection, and autoimmune diseases (3, 4). The clinical contribution of TLSs to diseases has garnered attention owing to the association of TLSs with a favorable response to immune checkpoint inhibitors in cancer tissues (5–7). In addition to cancers, autoreactive B cells capable of perpetuating autoimmune responses have been detected in TLSs of autoimmune diseases, such as rheumatoid arthritis and autoimmune thyroid diseases (8, 9).

Similar to B cell follicles in SLOs, TLSs have high expression of CXCL13, a chemokine responsible for the recruitment of CXCR5-expressing immune cells, such as naive B cells and T follicular helper (Tfh) cells (10). Although CXCL13 is commonly known as a chemokine produced by follicular dendritic cells in human SLOs and mice, CXCL13-producing T cells have been found in inflamed human tissues (11). Moreover, these CXCL13^+^ T cells are located in TLSs and contribute to their formation (12). Enrichment of CXCL13^+^ T cells predicts favorable outcomes to immunotherapy, indicating that T cells play a crucial role as CXCL13-producing cells (13). However, how CXCL13^+^ T cells are expanded and functionally regulated within TLSs is not yet known.

Here, we investigated TLSs containing DSG-specific B cells and plasma cells in chronic blisters from patients with pemphigus. We demonstrate upregulation of CXCL13, which is mainly produced by CD4^+^ T cells, in skin samples with TLSs. We also show that CXCL13^+^CD4^+^ T cells are clonally expanded. These CXCL13^+^CD4^+^ T cells are characterized by activated Th1-like CD4^+^ T cells, but paradoxically downregulated genes associated with T cell receptor (TCR) signaling when they had high expression of *CXCL13*. By using a highly multiplex imaging technique, we found that Tregs directly contact CXCL13^+^CD4^+^ T cells and promote the production of CXCL13 in CD4^+^ T cells. Finally, we report the decrease of TLSs and chronic blisters in patients with pemphigus after intralesional injection of corticosteroid. In conclusion, skin TLSs are associated with the maintenance of chronic blister in patients with pemphigus, and the microenvironmental network between clonal CXCL13^+^CD4^+^ T cells and Tregs is important for the production of CXCL13 in the TLSs.

## Results

### TLSs are commonly observed in the chronic skin lesions of patients with pemphigus.

**Figure 1:**
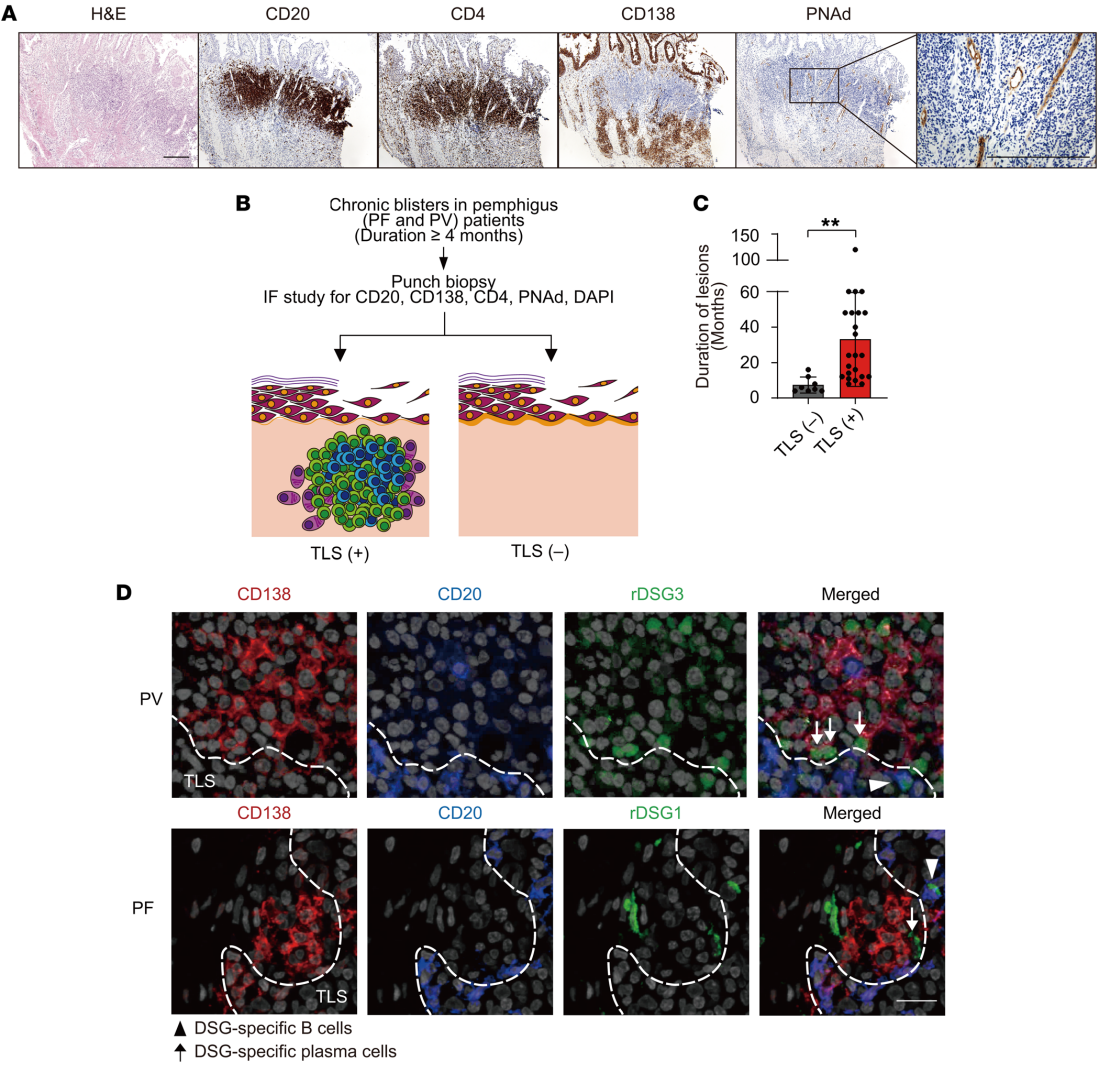
Skin tertiary lymphoid structures in chronic blisters of patients with pemphigus. (**A**) Skin biopsy sample showing tertiary lymphoid structures (TLSs) from a patient with PV (Supplemental Figure 1A, no. 3) stained with H&E and specific antibodies against CD20, CD4, CD138, and PNAd. Scale bar: 100 μm. (**B**) Schematic of the experiment. (**C**) Duration of skin blisters in TLS-negative (*n* = 8) and TLS-positive (*n* = 23) lesions. Student’s *t* tests were used to compare means for 2 groups. Data are shown as the mean ± SD. **P < 0.005. (**D**) Representative immunofluorescence staining for DSG-specific B cells (triangles) and plasma cells (arrows). Tissues were stained with CD138 (red), CD20 (blue), rDSG1 or rDSG3 (green), and DAPI (light gray). Dotted lines indicate the margin of TLSs. Scale bar: 50 μm. PF, pemphigus foliaceus; PV, pemphigus vulgaris; rDSG1, recombinant desmoglein 1; rDSG3, recombinant desmoglein 3.

We retrospectively reviewed H&E-stained slides of skin specimens from patients with pemphigus. Skin specimens with dense inflammatory cell infiltration into the dermis were selected from the slides. To analyze the phenotypes of immune cells infiltrating the dermis, we utilized immunohistochemistry to investigate expression of CD4, CD20, CD138, and peripheral lymph node addressin (PNAd). Tight clusters of CD4^+^ T cells and CD20^+^ B cells containing PNAd^+^ high endothelial venules surrounded by CD138^+^ plasma cells were observed in skin lesions from 6 patients with PV, 3 patients with PF, and 1 patient with PNP (Figure 1A and Supplemental Figure 1, A–C; supplemental material available online with this article; https://doi.org/10.1172/JCI166357DS1). These TLSs include follicular dendritic cells, CD11c^+^ dendritic cells, D2-40^+^ lymphatic vessels, and lymphotoxin β (Supplemental Figure 1D). These findings indicate that TLSs can be present in pemphigus skin lesions with dense inflammatory cell infiltration into the dermis.

Through examination of the clinical characteristics of the patients with TLSs, we found that the duration of skin lesions was at least 4 months (Table 1). To further analyze skin TLSs in pemphigus, we enrolled patients with pemphigus with chronic bullae persisting for more than 4 months, obtained punch biopsies of the chronic lesions, and carried out immunohistochemical studies for CD20, CD138, CD4, and PNAd to identify TLSs (Figure 1B and Supplemental Table 1). Of the 31 patients, 23 had skin samples positive for TLSs, whereas 8 were negative for TLSs (Figure 1B). More than half of the lesions with TLSs were located in the scalp (*n* = 13; Supplemental Table 1). The lesions with TLSs had a significantly longer duration than lesions without TLSs, and all skin TLSs were detected in blisters that persisted for at least 8 months (Figure 1C). When we investigated the antigen specificity in TLSs, we found that B cells and plasma cells specific for DSG3 and DSG1 were located at the edge of TLSs in samples from patients with PV and PF, respectively (Figure 1D). Taken together, these results confirm that TLSs with DSG-specific B cells and plasma cells are present in chronic blisters in patients with pemphigus.

### CD4^+^ T cells are the major CXCL13-producing cells in skin TLSs.

**Figure 2:**
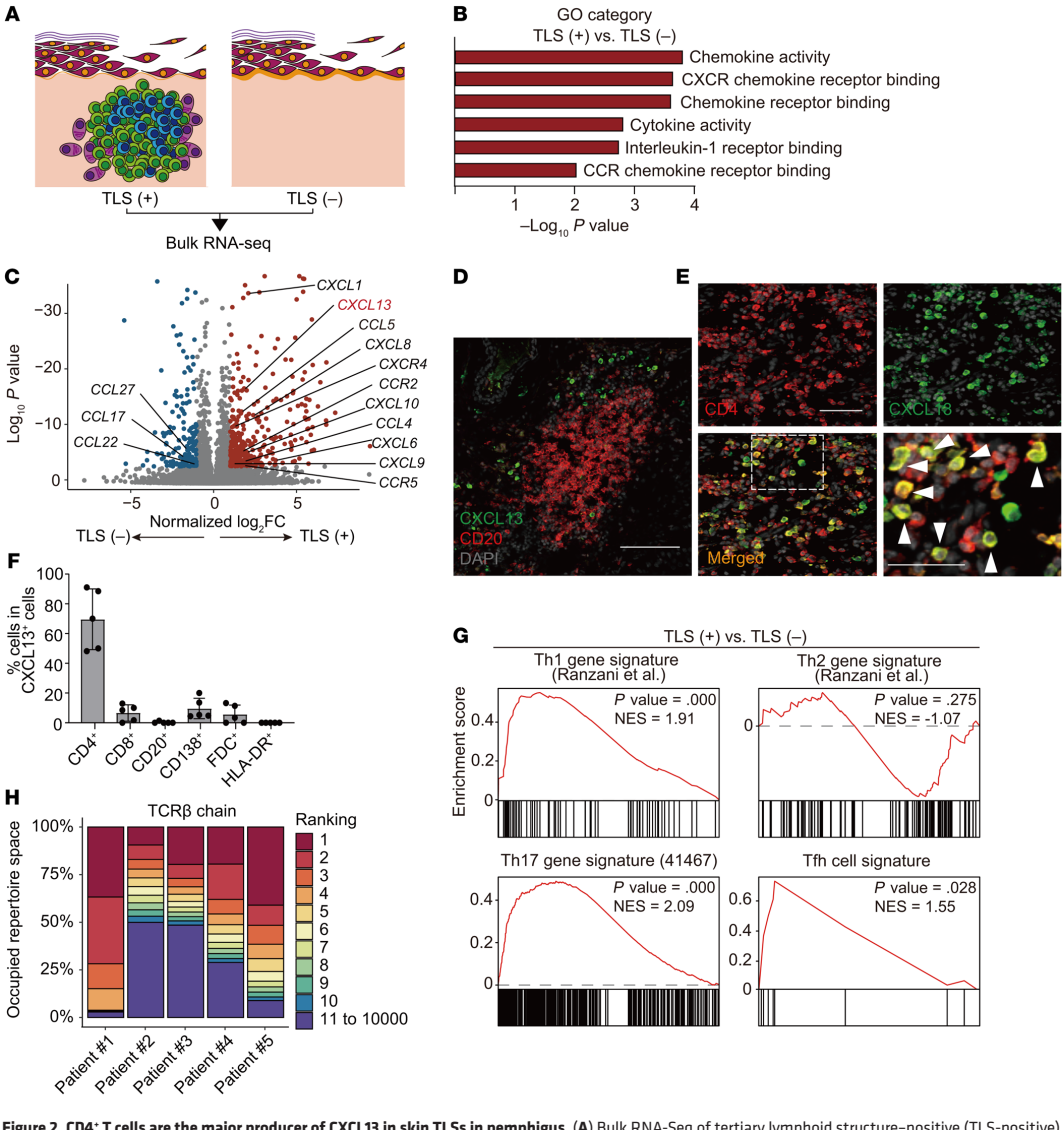
CD4^+^ T cells are the major producer of CXCL13 in skin TLSs in pemphigus. (**A**) Bulk RNA-Seq of tertiary lymphoid structure–positive (TLS-positive) and –negative samples (*n* = 5 each). (**B**) Gene ontology (GO) analysis and (**C**) volcano plot depicting upregulated (red dots) and downregulated DEGs (blue dots). (**D**) Representative immunofluorescence staining for CXCL13 (green) and CD20 (red) in skin TLSs from a patient with pemphigus. Nuclei were stained with DAPI (light gray). Scale bar: 100 μm. (**E**) Representative immunofluorescence staining for coexpression of CD4 (red) and CXCL13 (green) in skin TLSs. White arrowheads indicate CXCL13**^+^**CD4**^+^** cells. Nuclei were stained with DAPI (light gray). Scale bar: 50 μm. (**F**) Percentage of CD4^+^, CD8^+^, CD20^+^, CD138^+^, FDC^+^, and HLA-DR^+^ cells in CXCL13**^+^** cells from immunofluorescence images (*n* = 5). Data are shown as the mean ± SD. (**G**) Gene set enrichment analysis of Th1, Th2, Th17, and Tfh cell gene signatures using the transcriptome of TLS-positive versus TLS-negative samples. (**H**) Percentage of top 10 most frequently occurring clones of TCRβ chain from bulk TCR-Seq in skin TLSs from 5 patients (no. 1–5).

Next, we performed bulk RNA-Seq of pemphigus skin samples with TLSs (*n* = 5) and without TLSs (*n* = 5) and compared their gene signatures (Figure 2A). Consistent with the phenotypes, gene set enrichment analyses (GSEAs) showed that TLS-related gene signatures were upregulated in pemphigus skin lesions with TLSs compared with those without TLSs (Supplemental Figure 2A). Gene ontology (GO) term analysis revealed that chemokine activity and chemokine receptor binding genes were upregulated in skin lesions with TLSs compared with those without TLSs (Figure 2B and Supplemental Table 2). We identified 14 differentially expressed genes (DEGs) belonging to chemokine and chemokine receptor gene sets (Figure 2C, Supplemental Figure 2B, and Supplemental Table 3). Given that previous studies using conditional CXCL13-transgenic mice demonstrated that CXCL13 is sufficient to induce TLSs (14, 15), we focused on CXCL13 among the upregulated chemokines in skin lesions with TLSs. In immunofluorescence studies, CXCL13^+^ cells were mostly located in TLSs (Figure 2D). Next, we used various cell markers, including CD4, CD8, CD20, CD138, FDC, and HLA-DR, to identify CXCL13–producing cells (Supplemental Figure 3) and determined that the majority of CXCL13^+^ cells were CD4^+^ T cells (Figure 2, E and F). In GSEA, Th1, Th17, and Tfh cell gene signatures were enriched in skin lesions with TLSs, but the Th2 cell gene signature was not (Figure 2G). Bulk TCRβ RNA-Seq was performed to evaluate the distribution of TCR clones in skin lesions with TLSs. The top 10 clones constituted more than 50% of the total TCRβ repertoire (Figure 2H). These data suggest that T cells, including CXCL13^+^CD4^+^ T cells, in chronic skins with TLSs share TCR clones and have the characteristics of Th1, Th17, or Tfh cells.

### Clonal CXCL13^+^CD4^+^ T cells with Th1-like features are activated in skin TLSs.

**Figure 3:**
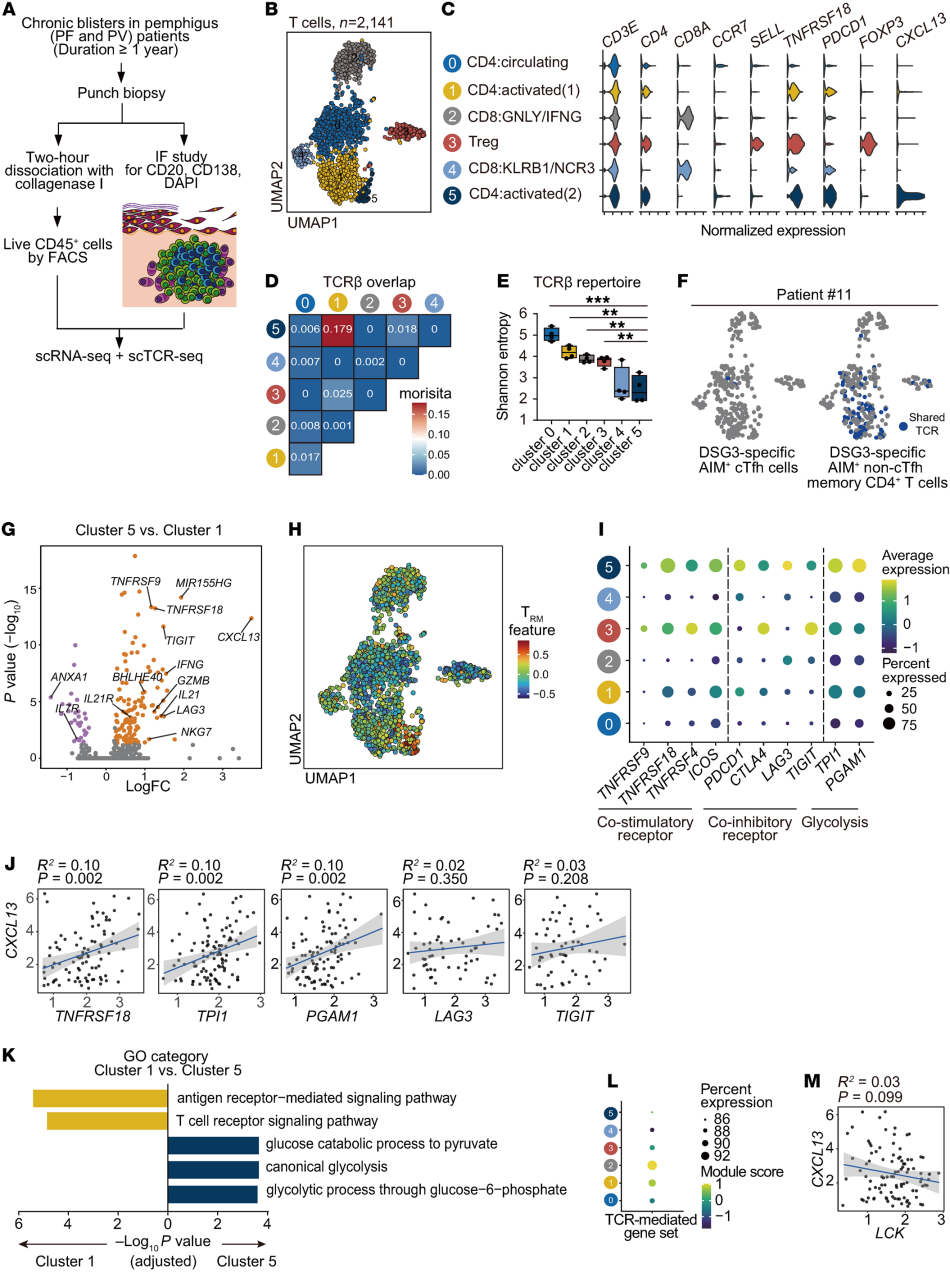
CXCL13^+^CD4^+^ T cells are clonally expanded and activated with Th1-like features. (**A**) Schematic of scRNA-Seq and scTCR-Seq for skin lesions with tertiary lymphoid structures (TLSs) in patients with pemphigus (*n* = 4). (**B**) UMAP visualization of 2,141 TCRβ^+^ T cells. (**C**) Violin plot showing the expression of the indicated marker genes in T cell subsets by scRNA-Seq. CD4, CD4^+^ T cells; CD8, CD8^+^ T cells. (**D**) Heatmap of the Morisita-Horn index quantifying overlapping TCRs among clusters. (**E**) The Shannon entropy calculation of the diversity of the TCR repertoire in each cluster. One-way ANOVA and Student’s *t* tests were used to compare means for 2 groups. **P < 0.005; ***P < 0.0001. Data are shown as the mean ± SD. (**F**) UMAP visualization of shared TCRs between T cells in skin TLSs and DSG3-specific, activation-induced marker (AIM)^+^ Tfh and non-Tfh memory CD4^+^ T cells in PBMCs from a patient with pemphigus vulgaris. (**G**) Volcano plot showing upregulated (orange dots) and downregulated (purple dots) in cluster 5 DEGs compared with cluster 1. (**H**) UMAP visualization showing gene signature of tissue-resident memory T (Trm) cells. (**I**) Dot plot showing expression of genes in the categories of costimulatory or coinhibitory receptors and glycolysis in each cluster. (**J**) Linear regression analyses of the expression of *CXCL13* and the correlation with expression of *TNFRSF18*, *TPI1*, *PGAM1*, *LAG3*, and *TIGIT* in clusters 1 and 5. Pearson’s correlation analysis was used to measure the strength of relationships between variables. (**K**) Gene ontology analysis in cluster 5 compared with cluster 1. (**L**) Dot plot depicting the TCR-mediated gene set in each cluster. (**M**) Linear regression analysis of the correlation between *CXCL13* and *LCK* in clusters 1 and 5. Pearson’s correlation analysis was used to measure the strength of relationships between variables.

Next, we performed single-cell RNA-Seq (scRNA-Seq) combined with single-cell TCR sequencing (scTCR-Seq) in samples from 4 chronic skin lesions that had lasted at least 1 year. Live CD45^+^ cells were sorted from most parts of the skin, and immunofluorescence studies confirmed the presence of skin TLSs using the remaining part of the skin (Figure 3A and Supplemental Figure 4A). After quality control and doublet exclusion (Supplemental Figure 4B), we obtained a total of 2,770 cells with 17 different clusters from all patient samples (Supplemental Figure 4C). To analyze T cells, we focused on 2,141 cells that were positive for TCRβ in 10 clusters (Supplemental Figure 4D).

We observed 6 clusters of TCRβ^+^ T cells in skin lesions with TLSs (Figure 3B). We further identified 3 clusters of CD4^+^ T cells expressing *CCR7* and *SELL,* corresponding to naive or circulating memory T cells, and 2 clusters of activated T cells expressing *TNFRSF18* encoding GITR (Figure 3C and Supplemental Table 4). Next, we evaluated TCR sharing and diversity in each cluster. Two activated CD4^+^ T cell clusters (clusters 1 and 5) shared their TCRs (Figure 3D and Supplemental Figure 4E), with cluster 5 being more clonal than cluster 1 (Figure 3E). Monocle 3 analysis showed that these 2 activated CD4^+^ T cell clusters are differentiated from 1 branch (Supplemental Figure 4F). To determine whether these clusters have DSG-specific autoreactive T cells, we identified the TCR repertoire in DSG3-reactive, activation-induced marker^+^ CD4^+^ T cells in PBMCs from 1 patient with PV (Supplemental Figure 4G) and matched TCRβ clones from the result to the TCR repertoire from scTCR-Seq from the same patient. We found that DSG-specific noncirculating Tfh (non-cTfh) memory CD4^+^ T cells, but not DSG-specific cTfh cells, were located in clusters 1 and 5 (Figure 3F). These data suggest that these 2 activated CD4^+^ T cell populations are clonally expanded.

To characterize CXCL13^+^CD4^+^ T cells, we compared the gene expression between 2 activated CD4^+^ T cell populations (Figure 3G and Supplemental Table 5). Analysis of DEGs revealed higher expression of *CXCL13* in cluster 5. We also found that cluster 5 had upregulated expression of *IFNG*, *BHLHE40*, and cytotoxicity-associated genes (*GZMB* and *NKG7*). Additionally, genes related to T cell activation (*TNFRSF9,* and *TNFRSF18*) and exhaustion (*LAG3* and *TIGIT*) were upregulated in cluster 5 (Figure 3G). Furthermore, the gene signature of tissue-resident memory T (Trm) cells was most highly expressed in cluster 5 (Figure 3H). To confirm that these features are consistent with those of CXCL13^+^CD4^+^ T cells, we divided them into CXCL13-expressing and CXCL13-nonexpressing groups in clusters 1 and 5 (Supplemental Figure 4, H and I). We found that CXCL13^+^ cells expressed genes similar to those of cluster 5 (Supplemental Figure 4J and Supplemental Table 6). Moreover, we observed that 38.1% of *CXCL13*-expressing cells also expressed the *IFNG* gene (Supplemental Figure 4I). As CXCL13^+^CD4^+^ T cells exhibited both an activated and exhausted phenotype, we further analyzed which phenotype was associated with the expression of *CXCL13*. We observed upregulation of glycolysis-associated genes (*TPI1* and *PGAM1*) in cluster 5 (Figure 3I). In cells from clusters 1 and 5, the expression of *TNFRSF18*, *TPI1*, and *PGAM1* positively correlated with the expression of *CXCL13* but not the expression of *LAG3* and *TIGIT* (Figure 3J). GO term analysis of cluster 5 compared with cluster 1 revealed that genes associated with cell cycle and glycolysis were upregulated, whereas genes associated with TCR binding were downregulated (Figure 3K and Supplemental Table 7). TCR-mediated genes were particularly downregulated in cluster 5, and *LCK* tended to negatively correlate with expression of *CXCL13* (Figure 3, L and M). Taken together, these data suggest that clonally expanded CXCL13^+^CD4^+^ T cells have features of Th1-like cells but downregulate genes associated with TCR-mediated signaling when the expression of *CXCL13* was highly expressed.

### CXCL13^+^CD4^+^ T cells are spatially adjacent to Tregs.

**Figure 4:**
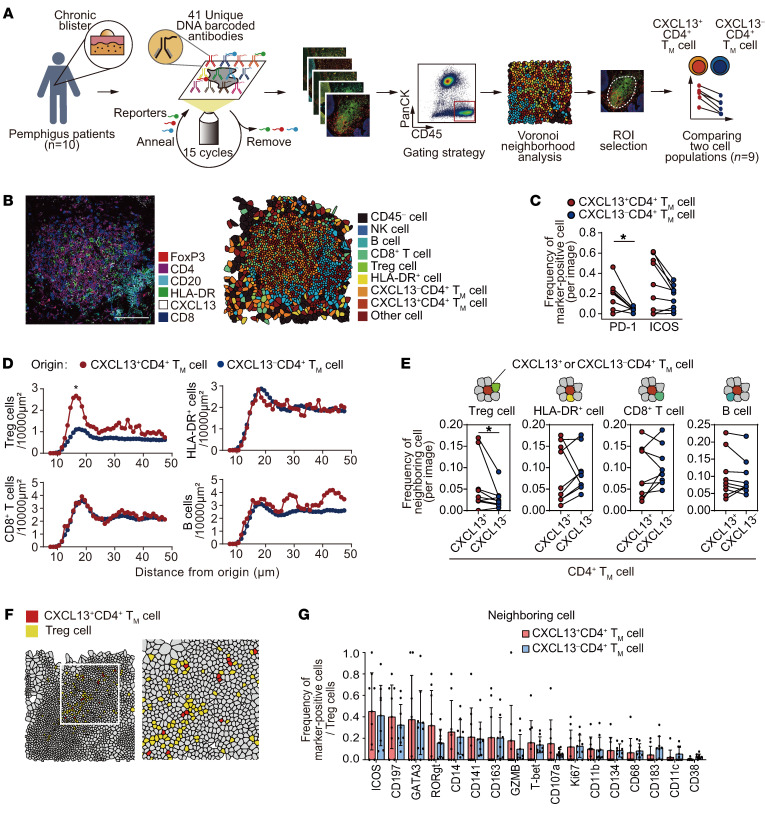
Tregs are adjacent to CXCL13^+^CD4^+^ T cells. (**A**) Workflow for CODEX imaging and analysis of skin tertiary lymphoid structures (TLSs) in patients with pemphigus (*n* = 9). (**B**) Six representative markers (left) for FoxP3 (red), CD4 (magenta), CD20 (cyan), HLA-DR (green), CXCL13 (white), and CD8 (blue) and a representative Voronoi diagram (right) of the TLSs after cell mapping. (**C**) Frequency of PD-1^+^ and ICOS^+^ cells in CXCL13^+^ versus CXCL13^–^ CD4^+^ Tm cells. Paired *t* tests were used to compare values for 2-variable plots. *P < 0.05. (**D**) Densities of Tregs, HLA-DR^+^ cells, CD8^+^ T cells, and B cells based on their distance from the center of CXCL13^+^ versus CXCL13^–^ CD4^+^ Tm cells. Wilcoxon matched-pairs signed-rank test. *P < 0.05. (**E**) Frequencies of Tregs, HLA-DR^+^ cells, CD8^+^ T cells, and B cells adjacent to CXCL13^+^ versus CXCL13^–^ CD4^+^ Tm cells in TLSs. Paired *t* tests were used to compare values for 2-variable plots. *P < 0.05. (**F**) Representative figures highlighting CXCL13^+^CD4^+^ Tm cells (red) and Tregs (yellow) in the Voronoi diagram. (**G**) Frequencies of marker-positive cells in Tregs adjacent to CXCL13^+^ versus CXCL13^–^ CD4^+^ Tm cells. Data are shown as the mean ± SD.

TCR stimulation induces expression of CXCL13 in CD4^+^ T cells in vitro (16). In contrast, we observed downregulation of genes associated with TCR signaling in CXCL13^+^CD4^+^ T cells within skin TLSs. To understand this phenomenon, we investigated the microenvironment and spatial organization of CXCL13^+^CD4^+^ T cells. In order to identify the cells that are spatially associated with CXCL13^+^CD4^+^ T cells, we employed a highly multiplex imaging technique, CO-Detection by indEXing (CODEX), with 41 protein markers to examine cell populations that interact with CXCL13^+^CD4^+^ T cells (Supplemental Table 8 and Supplemental Figure 5). We used formalin-fixed, paraffin-embedded tissues of chronic blisters from 36 TLSs in 10 patients with pemphigus to perform single-cell segmentation, assign cell type annotation by manual gating, visualize cell type using Voronoi diagrams to simplify the images, and analyze the data after selecting TLSs as ROIs (Figure 4A and Supplemental Figure 6A). We mainly investigated the immune cell types among T cells, including CXCL13^+^ and CXCL13^–^ cells in CD4^+^ memory T (Tm) cells, CD8^+^ T cells, and FoxP3^+^CD4^+^ Tregs. We also examined CD20^+^ B cells and HLA-DR^+^ cells (Figure 4B). After excluding 1 tissue that had fewer than 10 CXCL13^+^CD4^+^ Tm cells in an image, we compared immunophenotypes and neighboring cell frequencies between 2 cell types in 32 TLSs from the 9 images. CXCL13^+^CD4^+^ Tm cells had higher expression of PD-1 than CXCL13^–^CD4^+^ Tm cells (Figure 4C). In TLSs, we first computed the density of immune cell types (Tregs, CD8^+^ T cells, B cells, and HLA-DR^+^ cells) surrounding CXCL13^+^ and CXCL13^–^CD4^+^ Tm cells. Contrary to the data observed for CD8^+^ T cells, B cells, and HLA-DR^+^ cells, we observed a significantly higher density of Tregs located 16.5 μm away from the center of CXCL13^+^CD4^+^ Tm cells compared with their density around CXCL13^–^CD4^+^ Tm cells (Figure 4D). We further examined the frequencies of these immune cell types directly adjacent to CXCL13^+^ and CXCL13^–^CD4^+^ Tm cells. We found that the frequencies of Tregs adjacent to CXCL13^+^CD4^+^ Tm cells was significantly increased compared with that of CXCL13^–^CD4^+^ Tm cells (Figure 4, E and F). When analyzing the expression markers in neighboring cell populations, no difference was found between all neighboring cells, including Tregs, adjacent to CXCL13^+^ and CXCL13^–^CD4^+^ Tm cells (Figure 4G and Supplemental Figure 6B). Next, we utilized multiplex immunohistochemistry to analyze the spatial relationship between CXCL13^+^CD4^+^ T cells and CXCR5^+^ cells using specific markers for CD4, CXCL13, CD20, FoxP3, CXCR5, and PD-1 (Supplemental Figure 7A). Interestingly, we observed that CXCR5^+^PD-1^+^ Tfh cells and CXCR5^+^ B cells tend to be in proximity to CXCL13^+^CD4^+^ T cells, rather than being directly adjacent to them (Supplemental Figure 7B). Furthermore, with regard to Tregs adjacent to CXCL13^+^CD4^+^ T cells, we observed a higher frequency of CXCR5^–^ Tregs compared with CXCR5^+^ T follicular regulatory (Tfr) cells (Supplemental Figure 7C). Taken together, these findings indicated that Tregs are spatially adjacent to CXCL13^+^CD4^+^ T cells within the skin TLSs.

### Tregs increase the production of CXCL13 in CD4^+^ T cells.

**Figure 5:**
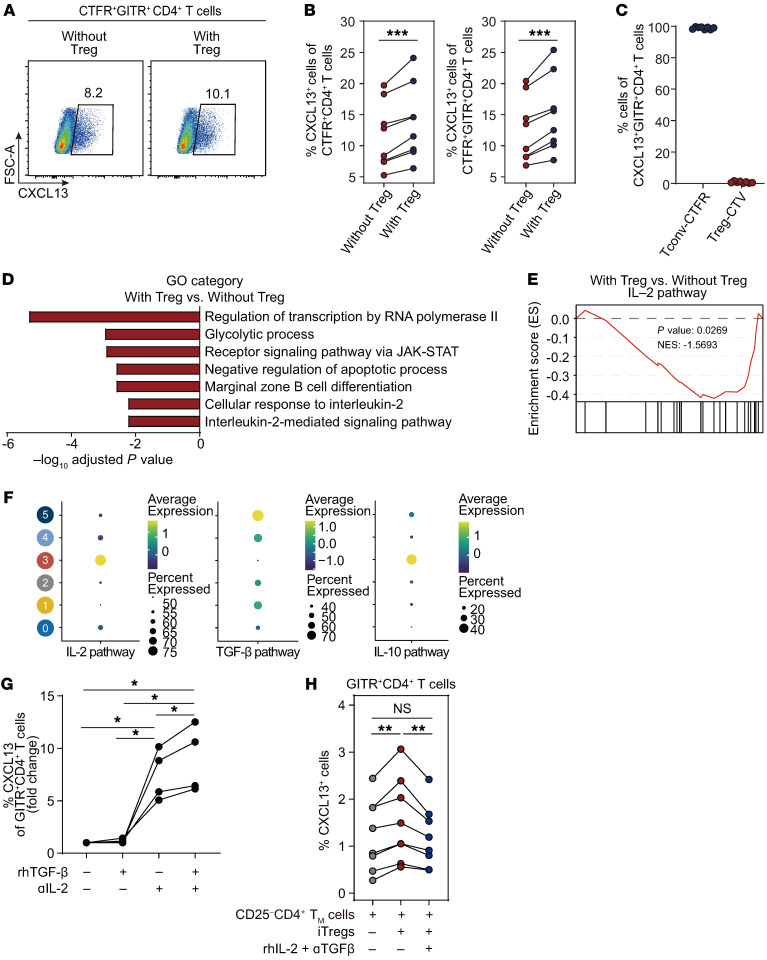
The production of CXCL13 in CD4^+^ T cells is increased by Tregs. (**A**–**F**) CXCL13^+^CD4^+^ T cells were differentiated in with and without Treg conditions in vitro. Conventional T cells were stained with CellTrace Far Red (CTFR), and sorted CD25^+^CD127^lo^CD4^+^ Tregs were stained with Cell Trace Violet (CTV). (**A**) Representative plots and (**B**) graph of the relative frequencies of CXCL13^+^ cells in CTFR^+^CD4^+^ and CTFR^+^GITR^+^CD4^+^ T cells (*n* = 8). Paired *t* tests were used to compare values for 2-variable plots. ***P < 0.0001. (**C**) Frequencies of CTFR^+^ conventional T cells and CTV^+^ Tregs in CXCL13^+^GITR^+^CD4^+^ T cells. (**D**) Gene ontology analysis using downregulated DEGs and (**E**) gene set enrichment analysis of IL-2 pathway gene signatures from the bulk RNA-Seq of CD4^+^ T cells in the Treg-undepleted condition compared with the Treg-depleted condition (*n* = 5). (**F**) Dot plot showing expression of genes involved in the IL-2 pathway, TGF-β pathway, and IL-10 pathway in each cluster, as assessed by scRNA-Seq. (**G**) CXCL13^+^CD4^+^ T cells were differentiated in the presence or absence of neutralizing anti–IL-2 antibody and/or TGF-β. Relative frequencies of CXCL13^+^ cells in GITR^+^CD4^+^ T cells (*n* = 5). Paired *t* tests were used to compare values for 2-variable plots. *P < 0.05. (**H**) Differentiated CXCL13^+^CD4^+^ T cells were cocultured with or without induced Tregs the in presence or absence recombinant IL-2 protein and TGF-β–blocking antibody. Relative frequencies of CXCL13^+^ cells in GITR^+^CD25^–/lo^CD4^+^ T cells (*n* = 8). Paired *t* tests were used to compare values for 2-variable plots. **P < 0.005.

To address whether and how Tregs regulate CXCL13 expression on CD4^+^ T cells, we used in vitro differentiation of CXCL13^+^CD4^+^ T cells (Supplemental Figure 8A). Based on our scRNA-Seq data showing upregulation of *TNFRSF9* and *TNFRSF18* in CXCL13^+^CD4^+^ T cells, we used 4-1BB and GITR as activation markers in our in vitro studies. Our results showed that approximately 30% of CXCL13^+^CD4^+^ T cells were 4-1BB^+^ cells, while almost 100% of CXCL13^+^CD4^+^ T cells were GITR^+^ cells (data not shown). Therefore, we selected GITR as a feasible marker to specifically evaluate the frequency of CXCL13^+^CD4^+^ T cells in activated T cells. Although the frequencies of IFN-γ^+^, TNF-α^+^, and IL-17A^+^ cells did not differ between the conditions of Treg presence and absence, we observed a reduction in the frequencies of CXCL13^+^ cells in both CD4^+^ T cells and GITR^+^CD4^+^ T cells in the absence of Tregs (Figure 5, A and B, and Supplemental Figure 8B). Tregs did not secrete CXCL13 in this condition (Figure 5C). To identify the factors regulating CXCL13 production, we compared bulk RNA-Seq profiles of CD4^+^ T cells after the differentiation of CXCL13^+^ cells in the presence or absence of Tregs. When evaluating GO term analysis and GESA, we found a significant decreased in gene expression involved in the IL-2–signaling pathway when differentiation occurred in the presence of Tregs (Figure 5, D and E, Supplemental Figure 8C, and Supplemental Table 9). In cluster 5 of the scRNA-Seq data, genes associated with the IL-2 pathway were downregulated and genes associated with TGF-β were particularly upregulated (Figure 5F). Next, we found that TGF-β and anti–IL-2–blocking antibodies synergistically increased CXCL13 expression in CD4^+^ T cells (Figure 5G). In vitro coculture of induced Tregs (iTregs) and differentiated CXCL13^+^CD4^+^ T cells (Supplemental Figure 8, D and E) resulted in increased CXCL13 production by CD4^+^ T cells, which was normalized when adding recombinant IL-2 protein and TGF-β–blocking antibody (Figure 5H and Supplemental Figure 8F). Taken together, these data suggest that Tregs increase CXCL13 expression in CD4^+^ T cells through IL-2 deprivation and TGF-β stimulation.

### Intralesional corticosteroid injection effectively controls chronic blistering with skin TLSs in patients with pemphigus.

**Figure 6:**
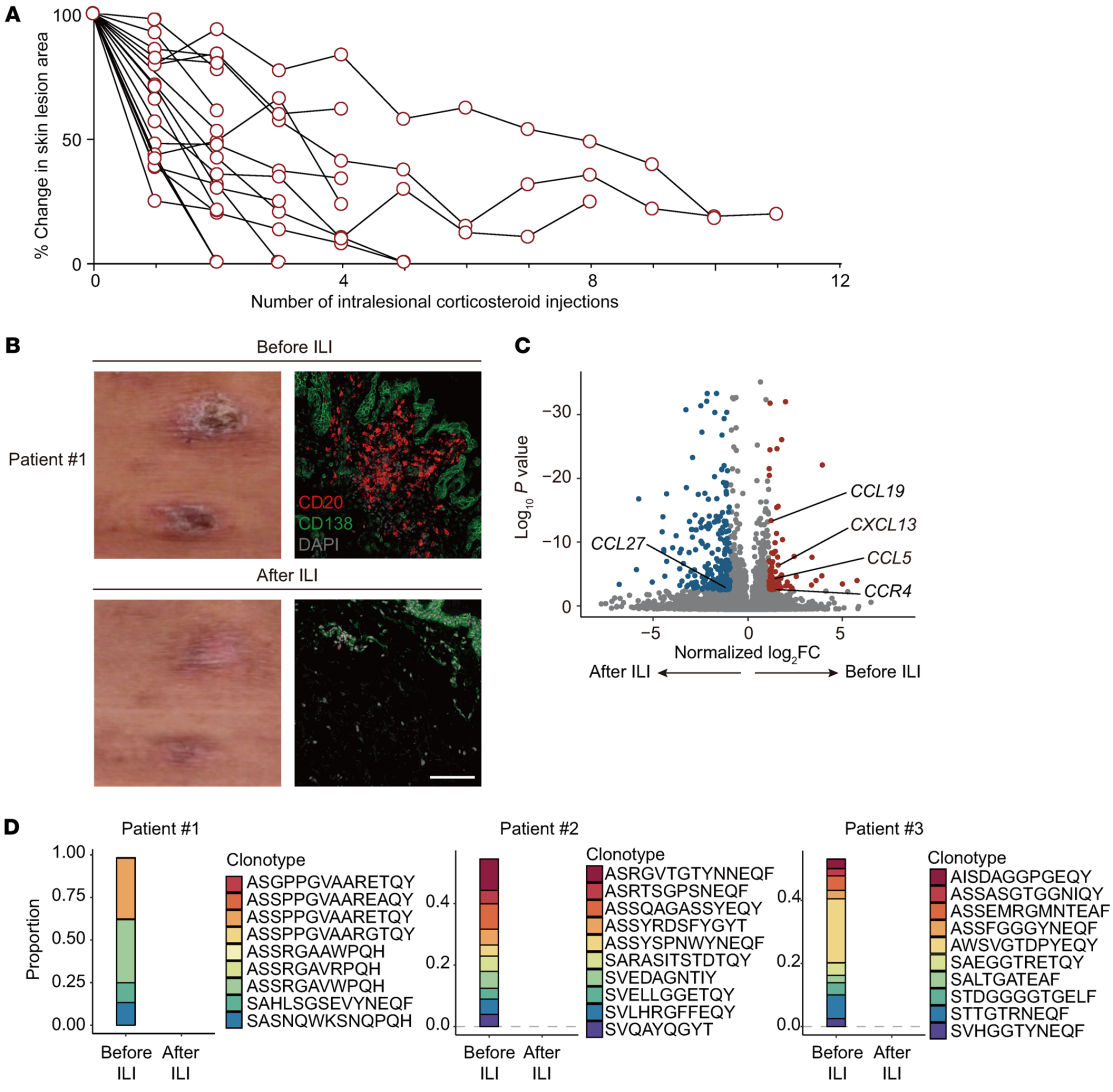
Intralesional corticosteroid injection ameliorates chronic blisters with skin TLSs in patients with pemphigus. (**A**) Change in skin lesion areas during Intralesional corticosteroid injection (ILI) in 18 patients. (**B**) Representative clinical images and immunofluorescence staining of a tertiary lymphoid structure–positive (TLS-positive) chronic lesion in patient 1 before and after ILI. Tissues were costained with CD20 (red), CD138 (green), and DAPI (light gray). Scale bar: 100 μm. (**C** and **D**) Bulk RNA-Seq of paired skin lesions with TLSs for comparison of before and after ILI (*n* = 3). (**C**) Volcano plot shows upregulated (red dots) and downregulated DEGs (blue dots). (**D**) Bulk TCR-Seq of paired skin lesions with TLSs for the comparison of before and after ILI (patient #1 to #3). The change in the proportion of top 10 clones of the TCRβ chains is shown for each patient.

We treated 18 patients with skin TLSs with intralesional corticosteroid injection (ILI). All lesions were reduced after treatment, and 5 of the lesions achieved complete clearance after ILI (Figure 6A). We observed the disappearance of TLSs in the removed lesion (Figure 6B). We obtained bulk RNA-Seq data for skin lesions with TLSs from 3 patients before and after ILI treatment and found that *CXCL13* was downregulated after treatment (Figure 6C and Supplemental Table 10). The top 10 TCR clones in lesions disappeared after ILI treatment (Figure 6D). Taken together, these results demonstrate that intralesional treatment with corticosteroids improves chronic blisters in pemphigus and reduces cutaneous TLSs.

## Discussion

The role of TLSs in the pathogenesis of pemphigus remains unknown, although TLSs have been detected in inflamed lesions in patients with pemphigus (17, 18). In this study, we showed local pathogenicity of skin TLSs in pemphigus. We observed that DSG-specific B cells and plasma cells colocalize in skin TLSs, suggesting that plasma cells that differentiate from DSG-specific B cells in TLSs produce pathogenic autoantibodies that contribute to suprabasal acantholytic blisters. Furthermore, our findings provide important evidence of the need for local treatment of chronic lesions in pemphigus, though the current treatment guidelines for pemphigus focus on systemic approaches based on the pathomechanism of B cell autoimmunity.

Given that mature TLSs provide an inflammatory antitumor environment and contribute to peripheral tissue autoimmunity (19–21), identification of factors that can control TLSs is crucial for treatment not only of cancers, but also autoimmune diseases. As CXCL13 is sufficient to induce TLSs (14, 15), it is important to understand the expansion and regulation of CXCL13-expressing cells in TLSs. CXCL13^+^ T cells in inflamed human tissues have various phenotypes depending on the disease (22, 23). In rheumatoid arthritis, CXCL13 is highly produced by PD-1^hi^CXCR5^–^CD4^+^ T cells in the synovium, which are different from Tfh cells (22). In malignancies, CXCL13^+^CD4^+^ T cells demonstrate high expression of *IFNG*, *GZMB*, and *PDCD1* (23). Moreover, PD-1^hi^CXCL13^+^CD8^+^ T cells have been detected in several cancers (24, 25). Consistent with these data, we observed that approximately 90% of CXCL13^+^CD4^+^ T cells in skin TLSs in pemphigus are PD-1^+^ cells. Furthermore, although demonstrated in only one sample, the activated CXCL13^+^CD4^+^ T cells include DSG-specific CD4^+^ T cells. Taken together, these data suggest that TCR stimulation may initially promotes the clonal expansion of CXCL13^+^CD4^+^ T cells.

Tregs have classically been recognized to suppress inflammation, but recent studies have shown that Tregs located in nonlymphoid tissues have functional diversity beyond immunosuppression (26). In terms of conventional Tfh cells, Tregs restrict their expansion mediated by CTLA-4 (27, 28). However, we observed paradoxical attenuation of genes associated with TCR signaling in CD4^+^ T cells expressing high levels of CXCL13, indicating that antigens are not necessary for high production of CXCL13 in CD4^+^ T cells within TLSs. Our results showed that Tregs directly contact CXCL13^+^CD4^+^ T cells and induce CXCL13 expression in CD4^+^ T cells. Furthermore, in agreement with previous studies (16, 29), we have shown that TGF-β and anti–IL-2–blocking antibody induce CXCL13^+^CD4^+^ T cells. TGF-β, especially, is known to enhance CXCL13 production in CD4^+^ T cells by increasing *SOX4* and repressing *SATB1* (16, 29). These data indicate the involvement of adjacent Tregs in the regulation of CXCL13^+^CD4^+^ T cells. Therefore, we suggest that Tregs play an important role in enhancing the secretion of CXCL13 in preexpanded clonal CD4^+^ T cells.

In the present study, the scalp was the most common site where skin TLSs were present, and skin TLSs, specifically B cell–enriched structures, were primarily located adjacent to hair follicles. Immune cells, including dendritic cells, various T cell subsets, and Tregs, have been known to be localized especially near hair follicles in the skin (30, 31), and hair follicles attract immune cells when under mechanical stress (32). This inflammation can occur during chronic autoimmune responses in the skin. Indeed, proinflammatory cytokines such as IL-6 and TNF-α also contribute to the generation of CXCL13^+^CD4^+^ T cells (16). Although CXCL13^+^CD4^+^ T cells may comprise a heterogeneous population of cells (33), our scRNA-Seq analysis revealed that the Th1-like Trm cell feature was prominently detected in these cells. Given that chronic lesions with skin TLSs tend to be persistent, CXCL13^+^CD4^+^ T cells, which possess skin-resident features, can potentially contribute to long-lasting blisters. It is possible that this characteristic is influenced by microenvironmental factors, such as IL-7 and IL-15, which are produced by the hair follicle (34). Taken together, preferential involvement of the scalp for skin TLSs may be due to the microenvironment promoted by high hair density.

In conclusion, our study showed the pathogenicity of skin TLSs and introduces a local therapeutic approach for controlling chronic lesions in pemphigus. Our approach provides critical insights byusing scRNA-Seq and scTCR-Seq data coupled with highly multiplex imaging data into the expansion and activation of CXCL13^+^CD4^+^ T cells as important drivers of TLS formation. Our findings may contribute to understanding the development of TLSs in other diseases, including cancers as well as autoimmune diseases.

## Methods

### Patients.

A total of 31 patients with pemphigus with chronic blisters were enrolled, including 18 who were treated with intralesional corticosteroids after the presence of skin TLSs in the lesions was confirmed (NCT04509570). The time period was established by obtaining a detailed patient history. Regarding cases of recurrence, lesion duration was defined as the period after by rituximab treatment of the last recurrence. Triamcinolone (10 mg/mL) was used each month until the lesions disappeared. Numbers of treatments varied in patients, and treatment was stopped when patients refused continuation of treatment or experienced relapse. Changes in the skin lesions were calculated using ImageJ (NIH).

### Immunohistochemistry.

For immunohistochemical staining, paraffin-embedded tissues were obtained from Human Tissue Bank of Gangnam Severance Hospital, Yonsei University College of Medicine, and were sectioned (5 μm) and stained with primary antibodies: rabbit anti-human CD138 (EP201), rabbit anti-human CD4 (SP35), rabbit anti-human CD11c (EP157), and mouse anti-human podoplanin (D2-40) (all from Cell Marque); mouse anti-human CD20 (L26) and rabbit anti-human lymphotoxin β (both from Abcam); mouse anti-human FDC (CAN.42, Thermo Fisher Scientific); and rat anti-human PNAd (MECA-79, BioLegend).

### Immunofluorescence.

Fresh tissues were obtained from the patients via 4–6 mm punch biopsy. Half of the tissues were fixed in 1% paraformaldehyde for 4 hours at room temperature and incubated in 20% sucrose overnight at 4°C. The tissues were sectioned (4 μm) and permeabilized with 0.5% Triton X-100 for 30 minutes. After washing with PBS, the slides were incubated in blocking solution (X0909, DAKO) for 1 hour. Tissues were stained overnight at 4°C using the following primary antibodies: mouse anti-human CD20 (L26, Abcam); 6X His-recombinant human DSG1 and 6X His-recombinant human DSG3 (both from Cusabio); goat anti-human CXCL13 (R&D Systems); rabbit anti-human CCL5 (P230E, Thermo Fisher Scientific); rabbit anti-human CD138 (EP201), rabbit anti-human FDC (CNA.42), and mouse anti-human CD8 (C8/144B) (all from Cell Marque); rat anti-human CD4 (YNB 46.1.8, Santa Cruz); and mouse anti-human HLA-DR (L243, BioLegend). Tissues were washed with PBS and incubated for 1 hour at room temperature with secondary antibody: Alexa Flour 488–conjugated goat anti-mouse and anti-rat antibodies; Alexa Flour 594–conjugated goat anti-rabbit, anti-mouse, and anti-rat antibodies; Alexa Flour 647–conjugated goat anti-mouse and anti-rat antibodies (all from Invitrogen); donkey anti-goat antibody (Jackson ImmunoResearch); and mouse anti-6X His-tag antibody (R&D Systems). Nuclei were stained with DAPI (Thermo Fisher Scientific). Fluorescence images were captured on an LSM 780 confocal microscope (Carl Zeiss).

### In vitro culture of CXCL13-producing CD4^+^ T cells.

PBMCs from healthy volunteers were separated by standard Ficoll-Paque (GE Healthcare) density gradient centrifugation. CD4^+^ T cells were isolated by MACS using a human CD4^+^ T cell isolation kit (Miltenyi Biotec) according to the manufacturer’s protocol. Isolated CD4^+^ T cells were differentiated at 37°C in a 5% CO_2_ atmosphere for 5 days in IMDM (Thermo Fisher Scientific) containing 10% fetal bovine serum (Welgene) and 100 units/mL penicillin and streptomycin (Thermo Fisher Scientific). For the depletion of Tregs, CD4^+^ T cells were stained for 10 minutes at room temperature using PerCP-Cy5.5–conjugated anti-CD4 (RPA-T4), PE-Cy7–conjugated anti-CD127 (A019D5), and APC-conjugated anti-CD25 (BC96) (all from BioLegend) and sorted by FACS Aria III (BD Biosciences) into CD25^+^CD127^lo^CD4^+^ Tregs and other CD4^+^ T cells. Tregs were labeled with Cell Trace Violet (Invitrogen) and other CD4^+^ T cells were labeled with CellTrace Far Red (Invitrogen). To generate CXCL13-producing CD4^+^ T cells, cells were stimulated with plate-coated 5 μg/mL anti-CD3 (OKT3, Invitrogen) and soluble 10 μg/mL anti-CD28 (CD28.2, BD Biosciences) antibodies in the presence of 2 ng/mL TGF-β1 (Cell Signaling Technology), 10 ng/mL IL-6 (Peprotech), and 10 μg/mL neutralizing anti–IL-2 antibody (R&D Systems). For the coculture system of CXCL13^+^CD4^+^ T cells and iTregs, presorted CD4^+^ T cells were stained with the following antibodies: FITC-conjugated anti-CCR7 (G043H7), PerCP-Cy5.5–conjugated anti-CD4 (RPA-T4), PE-Cy7–conjugated anti-CD45RA (HI100), and PE-CF594–conjugated anti-CD25 (BC96) (all from BioLegend) and APC-conjugated anti-CD127 (HIL-7R-M21) (BD Biosciences). Then, they were sorted using a FACS Aria III into CCR7^+^CD45RA^+^CD25^–^ naive CD4^+^ T cells and other CD25^–^CD4^+^ Tm cells. CD25^–^CD4^+^ Tm cells were used for differentiation of CXCL13-producing CD4^+^ T cells. To generate iTregs (>95% of CD4^+^ T cells), CD25^–^CD4^+^ naive T cells were cultured in 96-well plates coated with 5 μg/mL anti-CD3 (OKT3, Invitrogen) and soluble 10 μg/mL anti-CD28 (CD28.2, BD Biosciences) antibodies and stimulated with 5 ng/mL TGF-β1 (Cell Signaling Technology) and 50 U/mL IL-2 (Peprotech) in 10% IMDM (Thermo Fisher Scientific) for 5 days. After 5 days, CXCL13-producing CD4^+^ T cells (400,000/well) were mixed with iTregs at a 2:1 ratio for 24 hours in 10% IMDM (Thermo Fisher Scientific) with or without 50 U/mL IL-2 (Peprotech) and 10 μg/mL neutralizing anti–TGF-β1 antibody (R&D Systems). To stain CXCL13-producing CD4^+^ T cells, monensin (BD Biosciences) was added during the last 5 hours of coculture. To assess cytokine secretion, CXCL13-producing CD4^+^ T cells were stimulated with 50 ng/mL PMA and 1 μg/mL ionomycin. After 1 hour, cells were treated with monensin for 5 hours. Cells were harvested and stained with the following antibodies: BV510-conjugated anti-CD3 (UCHT1, BD Biosciences), APC-conjugated anti-CD127 (HIL-7R-M21, BD Biosciences), PerCP-Cy5.5–conjugated anti-CD4 (RPA-T4, BioLegend), FITC-conjugated anti-GITR (108-17, BioLegend), and PE-CF594–conjugated anti-CD25 (BC96, BioLegend). Dead cells were excluded using the LIVE/DEAD Fixable Red or Near-IR Cell Stain Kit (Invitrogen). For intracellular staining, the cells were fixed and permeabilized using the Foxp3/Transcription Factor Staining Buffer Set (Invitrogen). After permeabilization, cells were incubated for 30 minutes at 4°C with PE-conjugated anti-CXCL13 (IC801P, R&D Systems), BV605-conjugated anti–TNF-α (Mab11, BioLegend), BV711-conjugated anti–IFN-γ (B27, BD Biosciences), and BV786-conjugated anti–IL-17A (N49-653, BD Biosciences). Data were acquired using a BD FACS Aria III and analyzed with FlowJo software (BD Biosciences).

### Bulk RNA- and TCR-sequencing.

Total RNA was isolated from skin TLS-positive and -negative patients with pemphigus, TLS lesions before and after intralesional corticosteroid injection, and in vitro Treg-undepleted and Treg-depleted CXCL13-producing CD4^+^ T cells using TRIzol (Invitrogen) reagent following the manufacturer’s instructions. For bulk RNA-Seq, isolated total RNA was subjected to sequencing library production using the SureSelect RNA Direct kit (Agilent Technologies) for skin and the TruSeq Stranded mRNA Library Prep Kit (Illumina) for T cells according to the manufacturer’s protocol. Briefly, the cDNA library was created with a thermal cycler and the exon regions captured using SureSelect XT Human All Exon V6+UTRs Kit (Agilent Technologies) for skin and the SMART-Seq v4 Ultra Low Input RNA kit (Takara Bio Inc.) for T cells. The captured libraries were sequenced by Novaseq (Illumina). For bulk TCR-Seq, isolated total RNA was subsequently amplified using human TCR chain primers for TCR libraries, which were sequenced by Miseq (Illumina).

### Analysis of bulk RNA- and TCR-sequencing.

To identify DEGs (*P* < 0.001, fold change >2 or <–2) in the bulk RNA-Seq data, the R package DEGseq was used. From the list of DEGs, an analysis of the GO categories was performed using the web-based tool EnrichR (https://maayanlab.cloud/Enrichr/). For gene signature-specific analysis, the GSEA software was used. The reference gene sets of Th1, Th2, and Th17 cell and IL-2 pathway and gene sets of the Tfh cell related to human cancer TLSs (19). The matrix visualization and analysis software Morpheus was used to draw a heatmap. In bulk TCR-Seq data, the proportion of top 10 clones of the TCRβ chains was calculated using the R package immunarch.

### TCR sequencing of DSG3-specific T cells.

Cryopreserved PBMCs from a patient with pemphigus were thawed and rested for 4 hours at 37°C. Cells were stimulated with 5 μg/mL recombinant human DSG3 protein (Cusabio) for 20 hours at 37°C in a 5% CO_2_ atmosphere. After stimulation, cells were incubated for 20 minutes at room temperature with a biotinylated anti-human-CXCR5 (RF8B2), followed by staining with BV421-conjugated streptavidin (both from BD Biosciences). Cells were washed and incubated with the following antibodies: BV510-conjugated anti-CD3 (UCHT1), PE-Cy7–conjugated anti-PD-1 (EH12.1), and APC-H7–conjugated anti-CD45RA (HI100) (all from BD Biosciences) and FITC-conjugated anti-CCR7 (G043H7), PerCP-Cy5.5–conjugated anti-CD4 (RPA-T4), PE-conjugated anti-CD25 (BC96), and APC-conjugated anti-CD134 (ACT35) (all from BioLegend). Dead cells were excluded using the LIVE/DEAD Fixable Red Dead Cell Stain Kit (Invitrogen). CD134^+^CD25^+^ (activation-induced marker^+^) cTfh cells and non-cTfh memory CD4^+^ T cells were sorted using a FACSAria III cell sorter (BD Biosciences) and cDNA synthesized using the SMARTer Human TCR α/β profiling kit (Takara Bio Inc.) according to the manufacturer’s instructions. Purified TCR libraries were assessed and quantified using Bioanalyzer 2100 (Agilent Technologies). All libraries were pooled together for 1 run of Illumina MiSeq 2 × 300 bp sequencing.

### Single-cell RNA and TCR sequencing.

Skin tissue samples were obtained by 6 mm punch biopsy. After removal of subcutaneous fat, the tissues were chopped and digested with 50 mg/mL Collagenase 1A (Sigma-Aldrich) using a gentleMACS dissociator (Miltenyi Biotec) for 2 hours. After incubation, 1 mg/mL DNase I (Sigma-Aldrich) was added. Dead cells were excluded using the Live/Dead Fixable Near-IR Cell Stain kit (Invitrogen), and single cells were stained with BV421-conjugated anti-CD45 (HI30) and PE-conjugated anti-EpCAM (9C4) (both from BioLegend). Live CD45^+^EpCAM^–^ cells were sorted using a FACS Aria III cell sorter (BD Biosciences). scRNA-Seq and TCR-Seq libraries were generated using the Chromium single-cell 5′ Library kit V1.1, Chromium 5′ Gel Bead Kit V1.1, and Chromium V(D)J Human T cell Enrichment Kit (10X Genomics) following the manufacturer’s instructions. Libraries were constructed and sequenced at a depth of 20,000 reads per cell for RNA or 5,000 reads per cell for TCR using the HiSeq 4000 platform (Illumina).

### Single-cell RNA and TCR sequencing analysis.

All downstream analyses were performed using Cell Ranger 7.0 and the R package Seurat (v4.0.4). Outlier gene detection rates (nFeautre_RNA <300 and >14,000) and high mitochondrial transcript load (>10%) were filtered from the analysis. The data were normalized using Seurat’s logNormalize with a scale factor of 10,000. Data from individual samples were combined into a single expression matrix after scaling. Then, the cell cycle scores were set as variables to regress out. The Uniform Manifold Approximation and Projection (UMAP) algorithm was used to reduce and visualize dimensionality, followed by the construction of a clustering analysis. DEGs among clusters were detected by the Seurat function “FindAllMarkers.” Select functional DEGs in each cluster were visualized via stacked violin plots. Volcano plots of DEGs (*P* < 0.05, log_2_ fold change >0.25 or <–0.25) were applied to show the genes with upregulated or downregulated expression using ggplot2. For TCR analysis, we selected the TCRβ repertoire. Unique clones were defined as single clones and nonunique clones as shared clones. Cell trajectory and pseudo-time analysis was performed using the Monocle 3 R package (v1.3.1). To examine whether particular GO terms were enriched in certain gene sets, we carried out GO enrichment analysis using EnrichR. GO categories with adjusted *P* < 0.05 were considered significant. The Seurat AddModuleScore was utilized to analyze the gene signature expression of TCR-mediated signaling and Trm cells. The TCR-mediated gene set was composed of *CALM1*, *CALM2*, *CALM3*, *CD247*, *CD3D*, *CD3E*, *CD3G*, *ELK1*, *FOS*, *FYN*, *GRB2*, *HRAS*, *JUN*, *LAT*, *LCK*, *MAP2K1*, *MAP2K4*, *MAP3K1*, *MAPK3*, *MAPK8*, *NFATC1*, *NFATC2*, *NFATC3*, *NFKB1*, *NFKBIA*, *PIK3CA*, *PIK3CG*, *PIK3R1*, *PLCG1*, *PPP3CA*, *PPP3CB*, *PPP3CC*, *PRKCA*, *PRKCB*, *PTPN7*, *RAC1*, *RAF1*, *RASA1*, *RELA*, *SHC1*, *SOS1*, *VAV1*, and *ZAP70*. The gene set of Trm cells was composed of *CXCL13*, *CXCR6*, *IL23R*, *ITGAE*, *PDCD1*, *CD69*, *FABP4*, *FABP5*, *ID2*, *ID3*, *NR4A1*, *IL10*, *IL2*, and *RUNX3* (35). Biocarta_IL2_pathway, wp_IL10_antiinflammatory_signaling_pathway, and tgf-beta signaling pathway were used as the IL-2, TGF-β, and IL-10 pathway gene set, respectively.

### Multiplex immunohistochemistry staining and imaging.

4 μm Formalin-fixed, paraffin-embedded tissue sections were used for imaging. Slides were heated for at least 1 hour in a dry oven at 60°C. The slides were dewaxed with Leica Bond Dewax solution (Leica Biosystems), and antigen retrieval was performed with Bond Epitope Retrieval 2 (Leica Biosystems) for 30 minutes. Primary antibody incubation was performed for 30 minutes after blocking with 1× antibody diluent/block solution (Akoya Bioscience) followed by OPAL polymer HRP incubation for 10 minutes. Primary antibodies used included CD4 (EPR6855), CD20 (L26), FoxP3 (236A/E7), and PD-1 [EPR4877(2)] (all from abcam), CXCR5 (D6L3C, Cell Signaling Technology), and CXCL13 (R&D Systems). OPAL Polymer HRP anti-mouse were used for CD20, CXCR5, and FoxP3; anti-rabbit antibodies were used for CD4 and PD-1; and anti-goat were used for CXCL13. Visualization of the antigen was performed using tyramide signal amplification (Akoya Biosciences) for 10 minutes, and, to remove bound antibodies, slides antigen retrieval was performed with Bond Epitope Retrieval 1 (Leica Biosystems) for 20 minutes. The process from blocking to antigen retrieval was repeated for every antibody used. For counterstaining, nuclei were stained with DAPI (Thermo Fisher Scientific). Stained slides were imaged by using the Vectra Polaris Automated Quantitative Pathology Imaging System (Akoya Biosciences). Representative images for training were selected by the Penochart (Akoya Biosciences), and the negative and positive of each marker were trained using the inForm Image Analysis software (Akoya Bioscience) to validate the markers. Each single cell was segmented based on DAPI, and phenotyping was performed according to the expression and intensity of each marker. We designated CXCL13^+^CD4^+^ cells (CXCL13^+^CD4^+^ T cells), CXCR5^+^PD-1^+^CD4^+^ cells (Tfh cells), CXCR5^+^FoxP3^+^CD4^+^ cells (Tfr cells), CXCR5^–^FoxP3^+^CD4^+^ cells (CXCR5^–^ Tregs), and CXCR5^+^CD20^+^ cells (CXCR5^+^ B cells). We selected 3 ROI with all existing CXCL13^+^CD4^+^ T cells, Tfr cells, Tfh cells, and naive B cells. To analysis the spatial cell-to-cell distance, the distance between the 2 nearest cells was calculated using the nearest neighbor analysis, and the distances of each target cell from the nearest CXCL13^+^CD4^+^ T cells were measured.

### CODEX tissue staining and imaging.

For multiplex tissue staining and acquisition, formalin-fixed, paraffin-embedded samples were mounted on no. 1.5 coverslips. Multiplex tissue staining and acquisition were performed by Enable Medicine in accordance with previously published methods (36). Coverslips were imaged on an inverted fluorescence microscope (Keyence BX-810) using a Plan Apo 20x 0.75 NA objective (Nikon). The multiplex imaging cycles were performed on a CODEX (Akoya Biosciences). Raw CODEX data were processed by Enable Medicine using a cloud processing pipeline. Briefly, deconvolution and extended depth of field were computed for each *Z*-stack, and neighboring tiles and sequential cycles were computationally aligned and stitched together. Finally, background subtraction was performed by subtracting the linearly interpolated background signal between the first and final background acquisitions. Nuclear cell segmentation was performed using the Mesmer model from the DeepCell library (37). Nuclear segmentation masks were stochastically dilated by flipping pixels with a probability equal to the fraction of positive neighboring pixels. This dilation was repeated for 9 cycles for all CODEX data. Biomarker expression levels were computed from the mean pixel values within each segmentation mask for each cell. TLSs were manually annotated by drawing ROIs overlaid on the CODEX images using the Enable Medicine Portal. These ROIs were then used to filter cells for further downstream analysis. Downstream analysis was always restricted to cells filtered by ROI. Analysis was performed on a per-cell level, per-TLS level, or per-image level. Two different measures of spatial proximity were used in this study: (a) cells directly in contact and (b) nearest neighbors. To define cells directly in contact, we generated Voronoi tessellations from the centroids of the segmentation masks. Cells that shared an edge in the Delaunay dual of the Voronoi tessellation were defined as being in direct contact. Nearest neighbors were identified per cell type and defined using the Euclidean distance. Only cells within the same TLS were considered when calculating nearest neighbors. For certain biomarkers, manual gating was applied to define a threshold of positivity. Gating was performed on a per-image basis.

### Computing cell density from imaging data.

A moving-window density analysis was performed on the multiplexed tissue images to calculate the local density of target cells in the vicinity of individual CXCL13^+^ or CXCL13^–^CD4^+^ T cells with varying distances. Initially, after extracting cell position data from the ROIs, the subsequent the analyses were conducted on each ROI. A circle with a radius of 5 μm was initially positioned with its center on a selected cell. This circle was then expanded radially in increments of 1 μm, up to a maximum radius of 100 μm. The number of target cells present in each circle was counted, and the “edge-corrected” area of each circle was calculated by excluding the area of each circle that extended beyond the boundaries of the ROIs. To identify where the circle intersected with the ROI boundaries, the extract function from the terra package in R was used, customized for our specific application. Finally, target cell densities in “moving windows” were computed by sweeping ring-shaped regions called rings radially outward up to a maximum distance. Each ring was defined as the region between two circles with radii differing by a specified width. The area and cell count of each ring was then calculated by subtracting the area and cell counts of the inner circle from those of the outer circle, respectively, followed by the calculation of local densities of target cells.

### Statistics.

Data were statistically analyzed with Graphpad Prism Software version 9.2.0. Multiple comparisons were analyzed using 1-way ANOVA with Bonferroni’s post hoc test. Statistical comparisons were analyzed by using 2-tailed Student’s *t* test, 2-tailed paired *t* test, and Wilcoxon matched-pairs signed-rank test for 2 groups. Pearson’s correlation analysis was used to measure the strength of relationships between variables. *P* values of less than 0.05 were considered significant. All results are presented as mean ± SD. The significance of the difference between groups was analyzed as described in the figure legends.

### Study approval.

The patient study was approved by the IRB of Gangnam Severance Hospital (IRB no. 3-2018-0302 and IRB no. 3-2019-0191).

### Data availability.

All data are available in the main text or the supplemental materials. Values for all data points in graphs are reported in the Supporting Data Values file. Sequencing data are archived in the NCBI’s Gene Expression Omnibus database (GEO GSE242234).

## Author contributions

JHK and SCK conceptualized the study and designed the experiments. DH performed immunofluorescence and immunohistochemistry imaging experiments and analyzed the resulting data. AYL conducted experiments using flow cytometry and analyzed the data. TK and DYK analyzed bioinformatics data. HK, HBD, RP, and ATM performed experiment using highly multiplex imaging. CK, KP, and HK analyzed multiplex imaging data. JHK, JYC, MYC, and AS recruited participants and collected samples. JYC contributed to clinical data analysis. TGK, JHS, and HJK provided experimental support. ECS, MK, and EJC acquired funding for the project. DH, JHK, AYL, and TK prepared the figures and tables. JHK, DH, and AYL wrote and edited the manuscript.

## Supplementary Material

Supplemental data

ICMJE disclosure forms

Supplemental table 1

Supplemental table 10

Supplemental table 2

Supplemental table 3

Supplemental table 4

Supplemental table 5

Supplemental table 6

Supplemental table 7

Supplemental table 8

Supplemental table 9

Supporting data values

## Figures and Tables

**Table 1:**
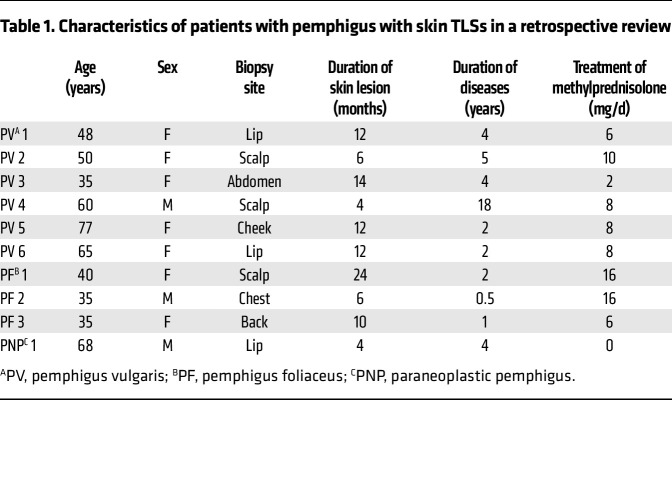
Characteristics of patients with pemphigus with skin TLSs in a retrospective review

## Version 1 10/10/2023

In-Press Preview

## Version 2 12/01/2023

Electronic publication
