# Supplementary material for: Microenvironmental network of clonal CXCL13+CD4+ T cells and Tregs in pemphigus chronic blisters
Source: J Clin Invest. 2023 Dec 1;133(23):e166357. doi: 10.1172/JCI166357 (PMC10688981; doi:10.1172/JCI166357)
Supplement: Supplemental data [file jci-133-166357-s106.pdf]

Supplemental Figure 1

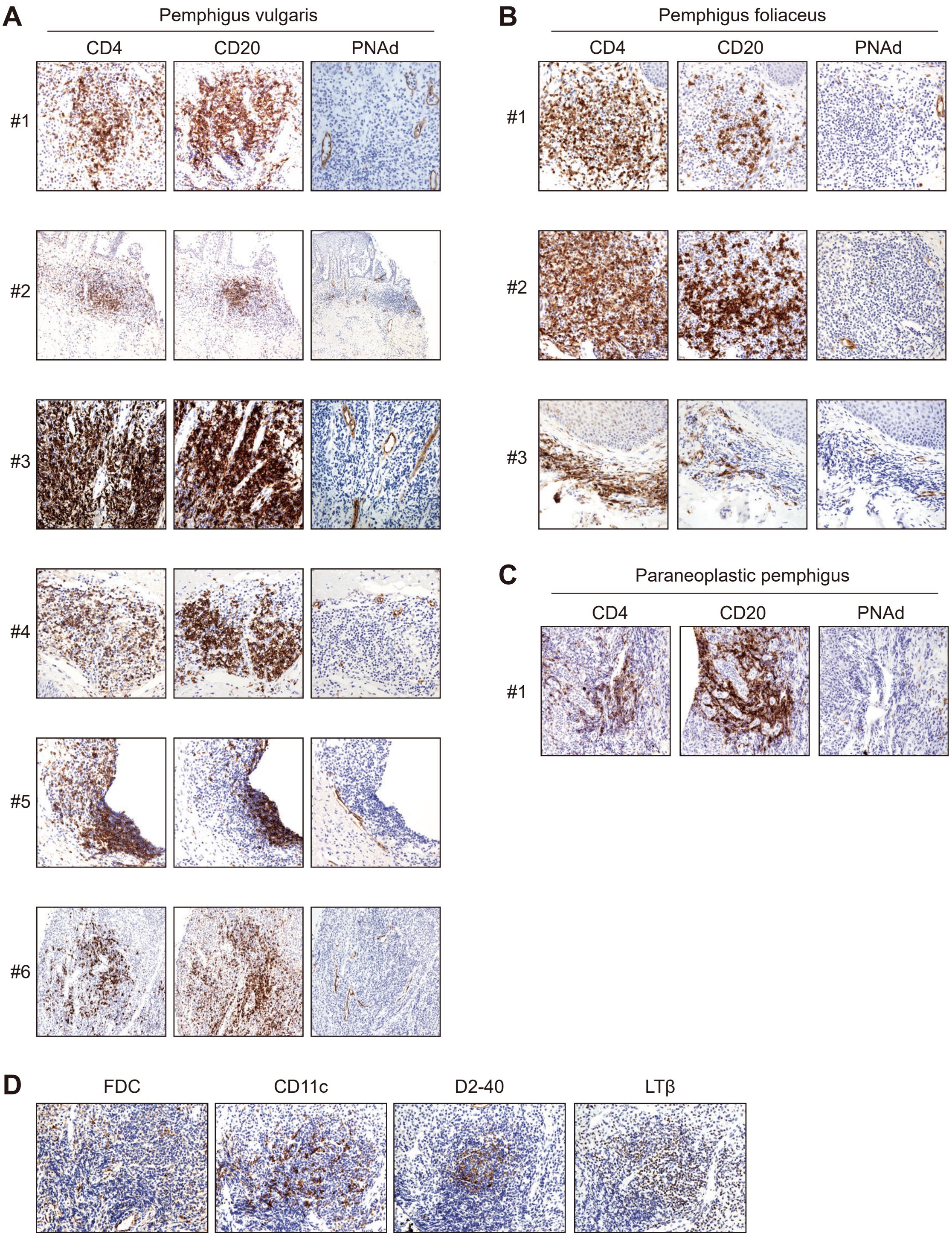

Supplemental Figure 1. Histological features of skin TLSs in pemphigus patients from the retrospective study. (**A-C**) Immunological staining for CD4, CD20, and PNAd in patients with pemphigus vulgaris (n=6) (**A**), pemphigus foliaceus (n=3) (**B**), and paraneoplastic pemphigus (n=1) (**C**). (**D**) Representative images of immunohistochemical staining for follicular dendritic cells (FDCs), CD11c, D2-40, and lymphotoxin β (LTβ).

Supplemental Figure 2

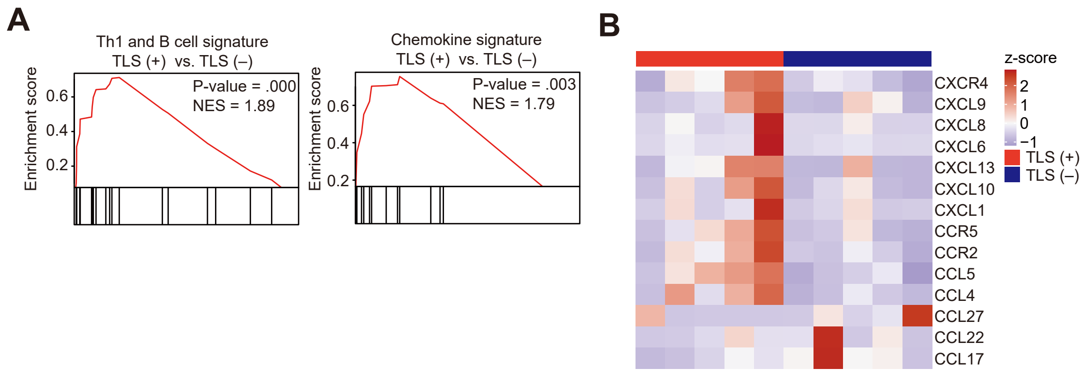

Supplemental Figure 2. Expression of gene signatures in TLS-positive and -negative cases. (**A**) GSEA of Th1 and B cell related signatures and chemokine gene signatures. (**B**) Heatmap showing gene expression of chemokine ligands and receptors.

Supplemental Figure 3

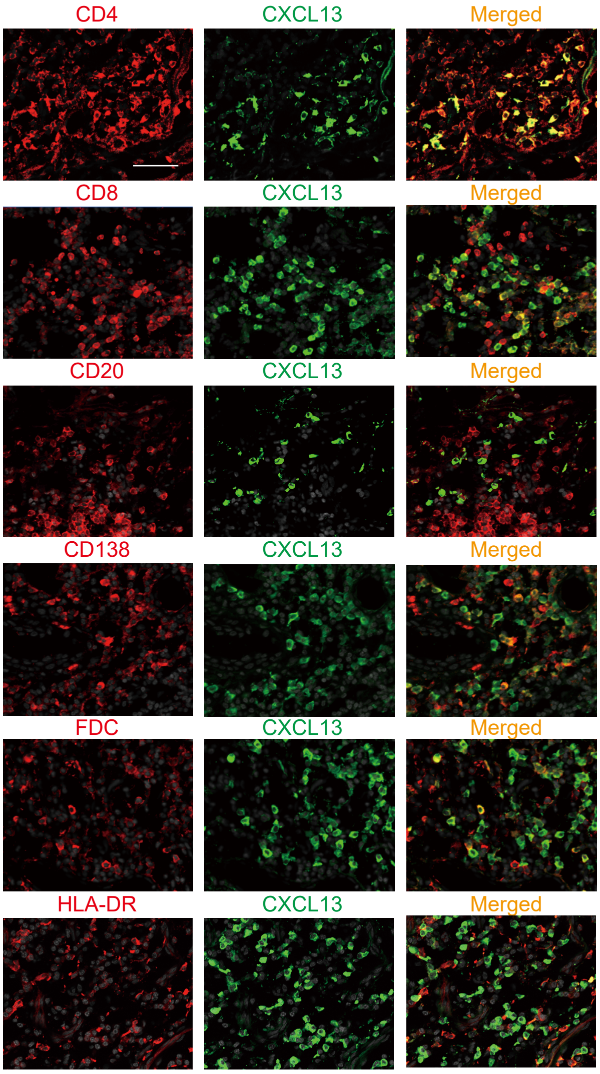

Supplemental Figure 3. Immunofluorescent staining of CXCL13^{+} cells in skin TLSs. Immunofluorescence of CXCL13 (green) with CD4, CD8, CD20, CD138, FDC, or HLA-DR (all red). Nuclei were stained with DAPI (light gray). Scale bar = 100 μm.

Supplemental Figure 4

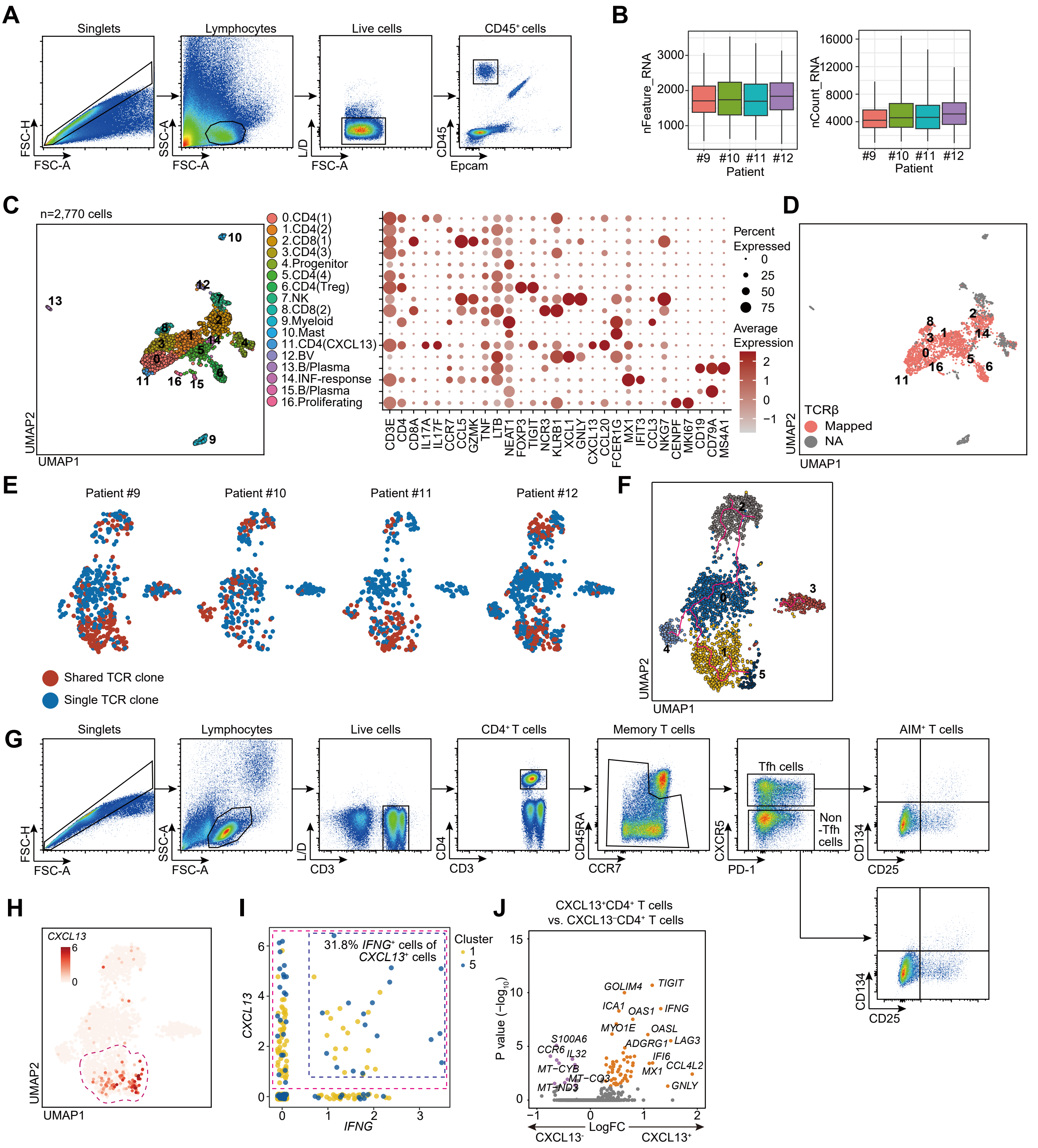

Supplemental Figure 4. scRNA-seq and scTCR-seq of chronic TLS skin lesions to characterize CXCL13⁺CD4⁺ T cells. (**A**) Gating strategy for flow cytometric analysis of live CD45⁺ cells in skin. L/D, Live/Dead. (**B**) Distribution of all expressed gene numbers and expressed genes in each sample. (**C** and **D**) UMAP visualization of 2,770 cells with 17 clusters in all patients (**C**) and TCRβ⁺ cells with 10 clusters (**D**). (**E**) Visualization of TCR clonality in each patient. Red dots indicate shared TCR clones and blue dots indicate single TCR clones. (**F**) Trajectory analysis of each cluster. (**G**) Gating strategy for flow cytometric analysis of CD134⁺CD25⁺ (AIM⁺) Tfh cells and non-Tfh memory CD4⁺ T cells in PBMCs from a patient with pemphigus vulgars. (**H**) *CXCL13* expression shown in UMAP. The red dotted line indicates clusters 1 and 5. (**I**) Expression of *CXCL13* and *IFNG* in clusters 1 and 5. The red dotted line indicates CXCL13⁺ cells and the blue dotted line indicates *IFNG*-expressing cells of CXCL13⁺ cells. (**J**) Volcano plot showing DEGs up-regulated (orange dots) and down-regulated (purple dots) in CXCL13⁺CD4⁺ T cells compared to CXCL13⁻CD4⁺ T cells.

Supplemental Figure 5

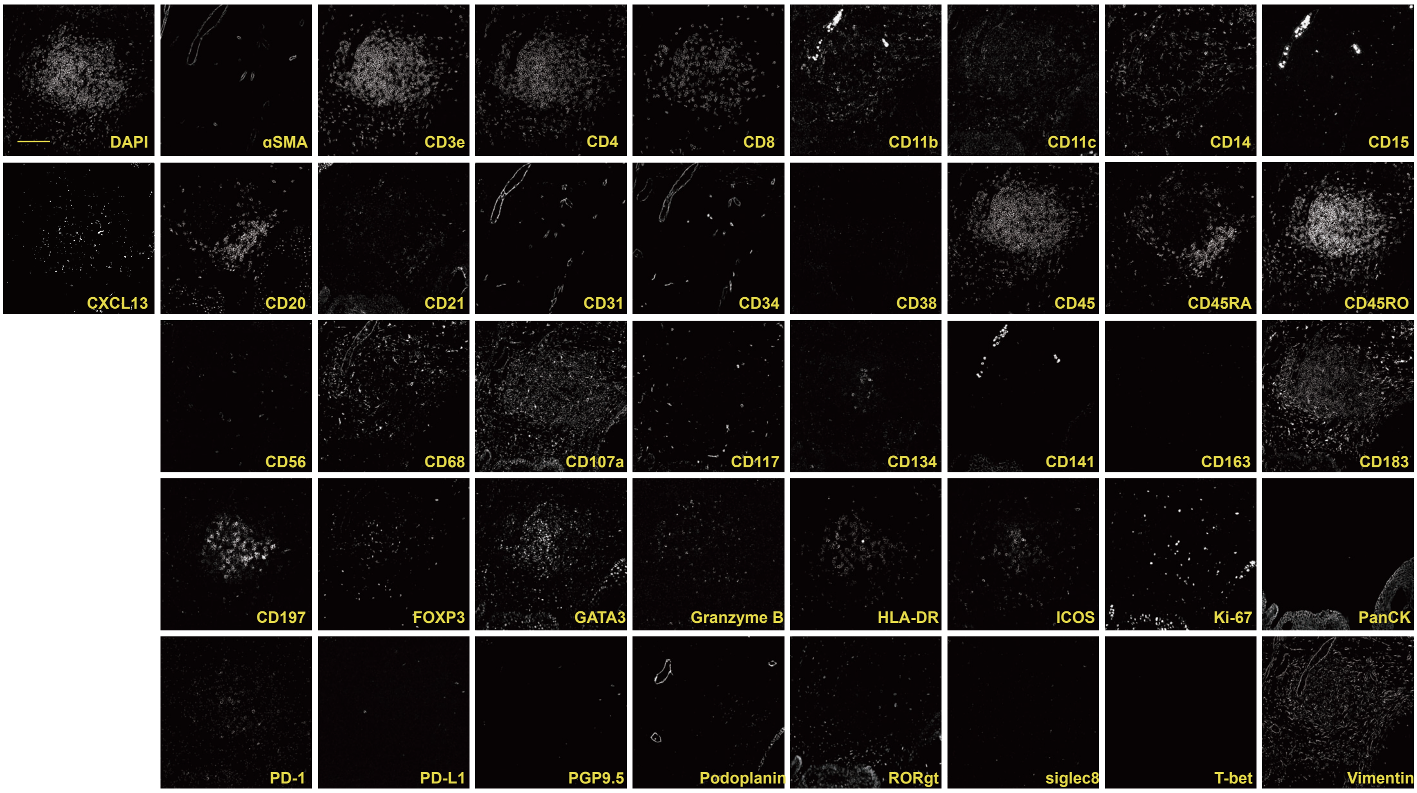

Supplemental Figure 5. Validation of the antibody panel in spatial proteomics. Representative images of a skin TLS stained with 41 different antibodies by CODEX. Scale bar = 100 μm.

Supplemental Figure 6

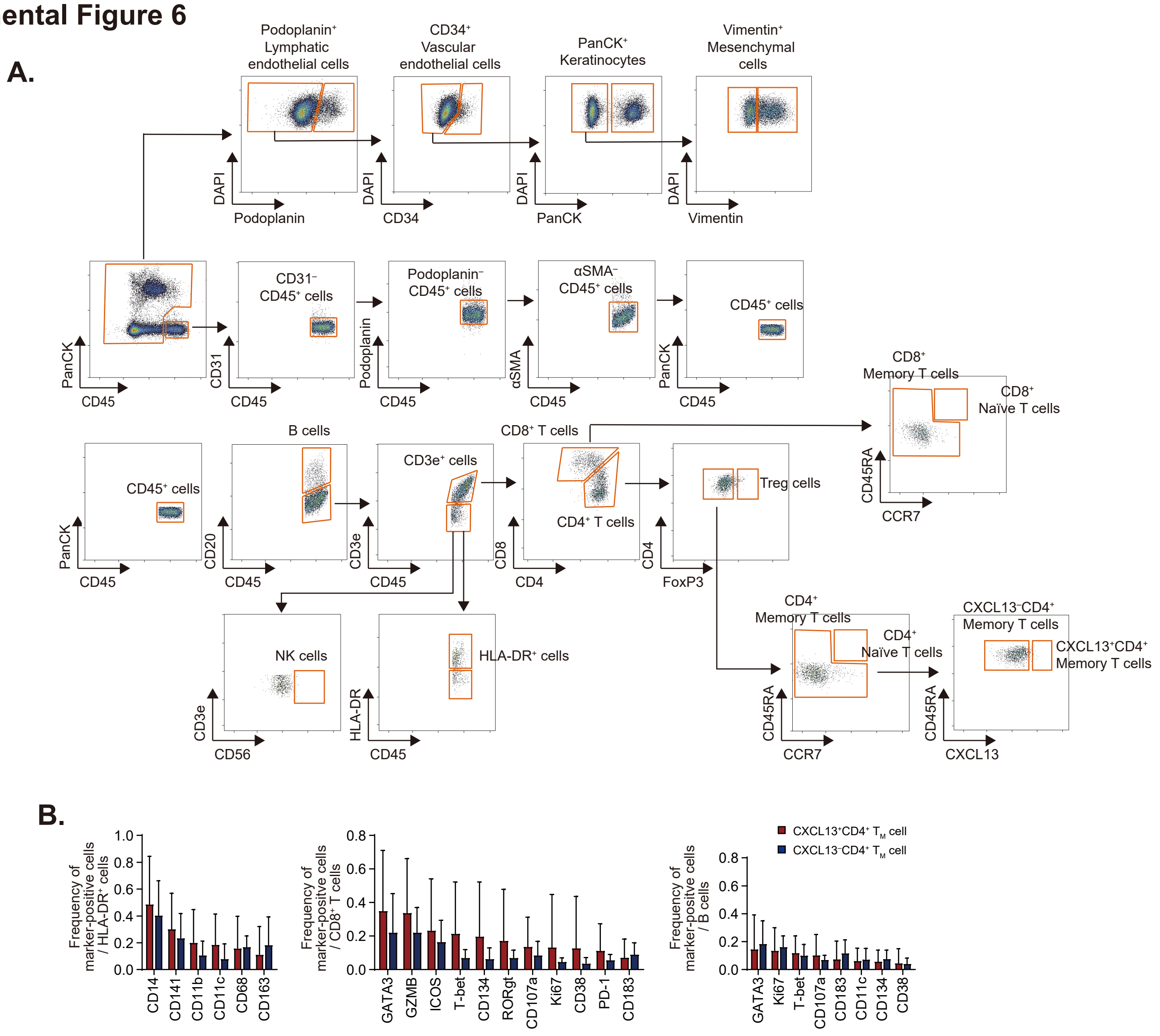

Supplemental Figure 6. Spatial analysis of CXCL13$^+$CD4$^+$ T cells. (**A**) Spatial proteomics gating strategy for cell type annotation. (**B**) Frequencies of marker-positive cells in CD8$^+$ T cells, HLA-DR$^+$ cells, and B cells adjacent to CXCL13$^+$ versus CXCL13$^-$ CD4$^+$ T$_M$ cells (n=9, per image).

Supplemental Figure 7

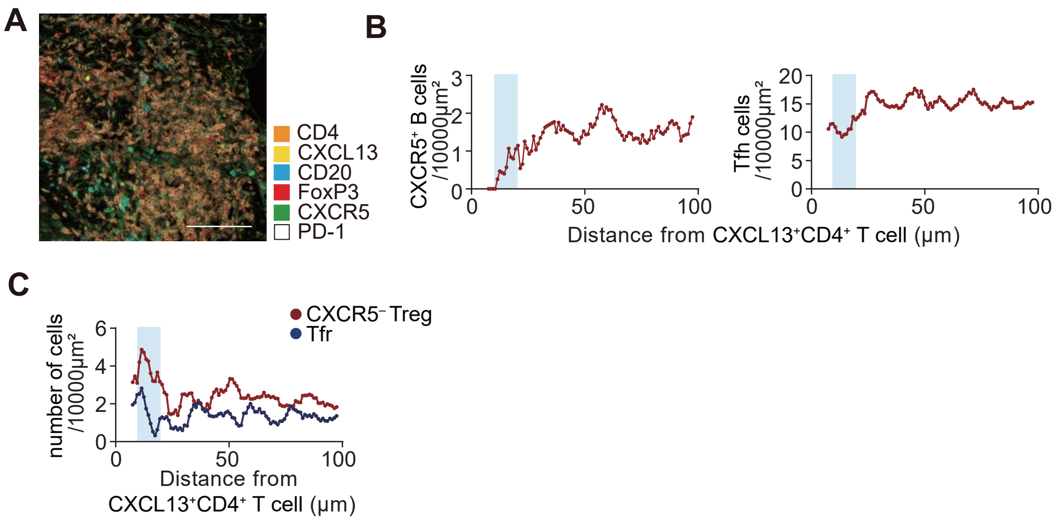

Supplemental Figure 7. mIHC analysis. (**A**) A representative image of mIHC by Vectra Polaris. Scale bar = 100 μm. (**B** and **C**) Densities of Tfh cells, $CXCR5^+$ B cells (**B**), $CXCR5^–$ Tregs, and Tfr cells (**C**) based on their distance from the center of $CXCL13^+CD4^+$ T cells. Number of ROI = 3. The highlighted blue regions are the location of 10-20 μm away from the center of $CXCL13^+CD4^+$ T cells.

Supplemental Figure 8

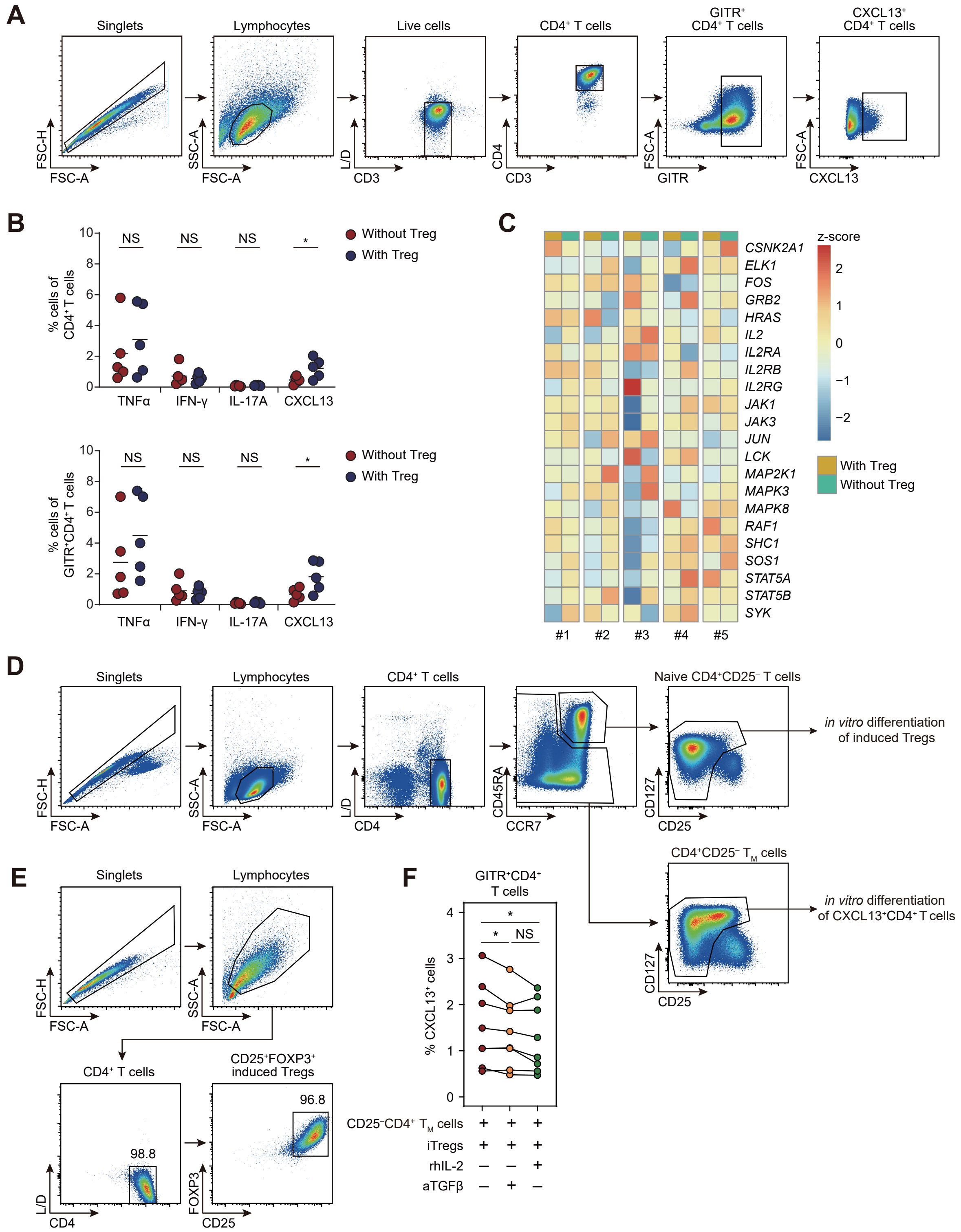

Supplemental Figure 8. Functional analysis of CXCL13⁺CD4⁺ T cells. (**A**) Gating strategy for flow cytometric analysis of CXCL13⁺CD4⁺ T cells *in vitro*. L/D, Live/Dead. (**B**) Graph showing the frequencies of IFN-γ⁺, TNF-α⁺, and IL-17A⁺ cells in the conditions of Treg presence and absence. A upper panel indicates percentage cells of CD4⁺ T cells and a lower panel indicates percentage cells of GITR⁺CD4⁺ T cells in the conditions of with and without Treg. Paired t tests were used to compare value for 2-variable plots. (**C**) Heatmap showing BiorCarta IL-2 pathway in with versus without Tregs condition. (**D** and **E**) Gating strategy for flow cytometric analysis of *in vitro* differentiation of CXCL13⁺CD4⁺ T cells (**D**) and induced Treg (**E**) for co-culture system. (**F**) Dfferentiated CXCL13⁺CD4⁺ T cells were co-cultured with induced Tregs in presence or absence recombinant IL-2 protein or TGF-β blocking antibody. Relative frequencies of CXCL13⁺ cells in GITR⁺CD25$^{-/low}$CD4⁺ T cells (n=8). Paired t tests were used to compare value for 2-variable plots. NS, not significant; *p < 0.05.
